# Unveiling the Association between HPV and Pan-Cancers: A Bidirectional Two-Sample Mendelian Randomization Study

**DOI:** 10.3390/cancers15215147

**Published:** 2023-10-26

**Authors:** Jianxuan Sun, Jiacheng Xiang, Ye An, Jinzhou Xu, Yifan Xiong, Shaogang Wang, Qidong Xia

**Affiliations:** Department and Institute of Urology, Tongji Hospital, Tongji Medical College, Huazhong University of Science and Technology, No. 1095 Jiefang Avenue, Wuhan 430030, China; sunjianxuan123@126.com (J.S.); jiachengxianghust@163.com (J.X.); ay121253@163.com (Y.A.); jason980620@163.com (J.X.); u201910348@hust.edu.cn (Y.X.)

**Keywords:** human papillomavirus, pan-cancer, HPV prevalence, Mendelian randomization

## Abstract

**Simple Summary:**

The association between HPV and cancer has been the focus of research, but there has been a lack of a comprehensive high-level evidence studies to systematically examine the relationship between them. Using Mendelian randomization, this paper provided an extensive analysis of the causal effect of HPV in cancer development. Our study conclusively identified HPV16 as a risk factor implicated in the development of bladder cancer, colorectal cancer, and breast cancer, while HPV18 was identified as a risk factor for prostate cancer, ovarian cancer, lung cancer and breast cancer. The results of Mendelian randomization also showed that HPV16 may be a protective factor for prostate cancer, anal cancer, lung cancer and oropharyngeal cancer, while HPV18 may be a protective factor for vaginal cancer.

**Abstract:**

Introduction: More and more studies have focused on the associations between human papillomavirus (HPV) infection and pan-cancers. However, current evidence is largely based on retrospective studies, which are susceptible to confounding factors and do not enable the establishment of causal relationships. Methods: A bidirectional two-sample Mendelian randomization (MR) design was employed to thoroughly evaluate the causal relationships between HPV and 12 site-specific cancers except cervical cancer. Single nucleoside polymers (SNPs) with strong evidence from genome-wide association studies (GWAS) were selected from HPV exposure datasets and used as instrumental variables (IVs) in this study. For the MR analysis results, MR-Egger’s intercept P test, MR-PRESSO global test, Cochran’s Q test and a leave-one-out test were applied for sensitivity analysis. Using HPVTIMER, we also performed immune infiltration analyses in head and neck squamous cell carcinoma (HNSCC), oropharyngeal squamous cell carcinoma (OPSCC) and vulval squamous cell carcinoma (VSCC) to evaluate the tumor-immune microenvironment. Results: Based on the evidence of MR analysis, our study conclusively identified HPV16 as a risk factor implicated in the development of bladder cancer, colorectal cancer, and breast cancer, while HPV18 was identified as a risk factor for prostate cancer, ovarian cancer, lung cancer and breast cancer. The MR results also showed that HPV16 may be a protective factor for prostate cancer, anal cancer, lung cancer and oropharyngeal cancer, while HPV18 may be a protective factor for vaginal cancer. Conclusion: An HPV infection may modulate the immune microenvironment and therefore has a potential inhibitory effect on the development of certain cancers. These conclusions provided new insights into the potential mechanisms of carcinogenesis and needed further research for validation.

## 1. Introduction

Human papillomaviruses (HPVs) are an ancient group of small DNA viruses, which wildly exist in nature and infect the mucosa or skin of vertebrates, including humans. HPV infection is considered as the most prevalent sexually transmitted infection worldwide [1]. It has been reported that around 45% men between 18 and 59 years old had a genital HPV infection in the United States [2], and about 80% of women of reproductive age have a lifetime risk of HPV infection [3]. About 450 types of human HPVs, which belong to five phylogenetic genera have been identified to date [4]. Among them, many types of HPVs can be regarded as normal microbial skin flora and only cause asymptomatic infections. Studies also demonstrated that high levels of beta-HPV found on male external genitalia [5], and detection of HPV DNA was reported in chronic otitis media and normal middle ear [6]. While other HPVs that cause lesions are artificially classified into two groups (high risk and low risk) according to their oncogenic potential. Low risk HPV (lrHPV), such as HPV 6 and HPV 11, cause Heck’s disease (oral focal epithelial hyperplasia) or genital warts depending on their infection sites [7]. High risk HPV (hrHPV), such as HPV16 and HPV18, cause intraepithelial neoplasia which can progress to cancer. It is estimated that 5.2% of cancers worldwide were ascribed to HPV infection [8]. In this context, identifying the relationship between HPV infection and the development of different cancers is particularly crucial.

A paradigmatic example of HPV-induced cancer is cervical cancer. The large majority of cervical cancer is attributed to HPV infection, in which HPV16 and 18 cause about 70% of cervical cancer in women worldwide [9]. A cervical hrHPV infection contributes to the presence of abnormal cervical cells. After 2 years, only 10% of infections still exist and become chronic persistent infections, and, at this moment, patients are at increased risk of cervical intraepithelial neoplasia (CIN) progression [10]. Suffering CIN1, CIN2 and CIN3 in succession, about one third of CIN3 lesions will progress to cancer within 10 to 20 years. In the process of lesion progression, earlier intervention leads to better prognosis. Extended evidence clearly demonstrated that HPV vaccination reduced the incidence of pre-cancer lesions, thus reducing the incidence of cervical cancer [11]. Except for cervical cancer, accumulated evidence shows that in some other solid tumors, such as vulvar cancer, penile cancer, anal cancer and head and neck cancers, hrHPV infection is predominantly involved in the neoplastic processes, and some patients are significantly associated with higher prevalence of HPV [12,13]. However, limited by the diversity of detection methods and sample types, there exists heterogeneity among current research. Meanwhile, the causal relationship between HPV infection and other solid tumors was still controversial. Cutaneous squamous cell carcinoma (cuSCC) is an important part of non-melanoma skin cancers, and ultraviolet radiation exposure is a confirmed risk factor for cuSCC. Although some studies reported a higher prevalence of HPV in cuSCC than normal people, HPV may not be responsible for the incidence of cuSCC, due to the natural accumulation of HPV in sun-exposure skin [14]. The debate on the relationship between breast cancer and HPV infection has been persistently existing for over 20 years. Nevertheless, there still lacks a unified conclusion [15]. Additionally, the association between HPV and urinary cancers, like bladder cancer and prostate cancer, also needs further verification. Recently, research on HPV-associated cancers have progressed rapidly, especially in the area of immune-based combination therapy. Meanwhile, therapeutic vaccines for HPV-related cancers have begun to conduct clinical trials. [16]. Therefore, a new and comprehensive analysis on HPV infection and these cancers is urgently needed to enhance the understanding of the relationship between HPV and cancers.

Mendelian randomization (MR) is an epidemiological method which uses single nucleotide polymorphisms (SNPs) as instrumental variables to clarify the casual relation between exposure and outcome. By utilizing genome-wide association studies instead of individual data, MR excludes the common invention of confounding factors in normal observational studies [17]. Moreover, using MR, we could exclude the potential bias of different detection methods and sample types through the detection process of HPV. Therefore, MR is an ideal method to evaluate the casual relation between HPV infection and cancer.

In this article, we conducted a comprehensive analysis on HPV infections and the development of cancers by MR. Indeed, cancers with a controversial relation between HPV infection were the main foci for us.

## 2. Materials and Methods

### 2.1. Study Design

**Figure 1:**
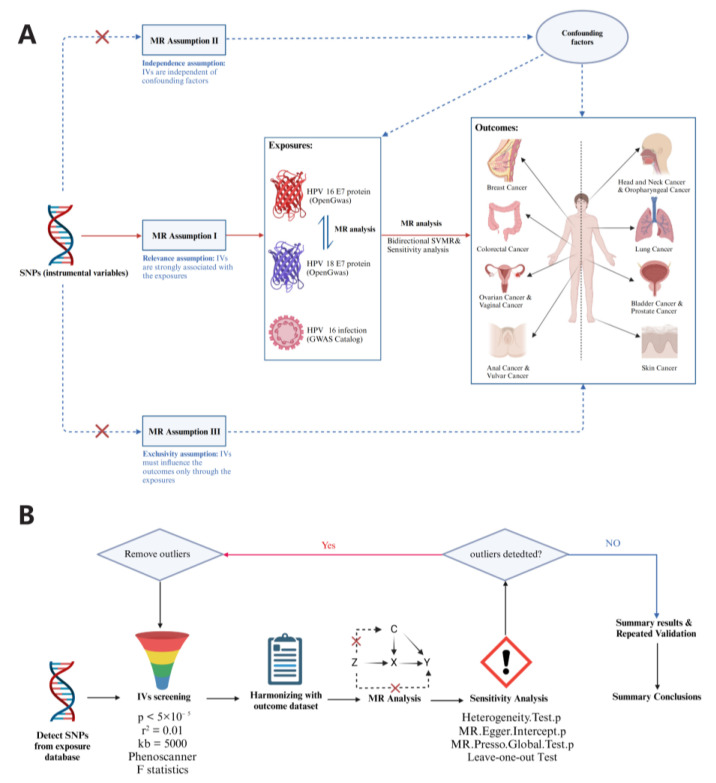
Overview of study design and analysis strategy. (**A**): Overview of study design. Exposures were from three datasets including HPV16 E7 protein, HPV18 E7 protein and chronic HPV infection. The MR framework was based on the three basic MR assumptions. (**B**): Analysis strategy of MR. Qualified SNPs were filtered as IVs and then subjected to sensitivity analyses and assessment of heterogeneity and pleiotropy. The results of the discovery and replication phases were summarized. In the MR analysis section, Z represents instrumental variables, X represents exposure factor, Y represents outcome factor, and C represents confouding factors. Note: MR: Mendelian randomization; HPV: human papillomavirus; SNP: single nucleotide polymorphism; SVMR: single-variable MR.

In this study, a two-sample Mendelian randomization [18] approach was adopted to assess the potential causal relationship between HPV-related indicators and cancer development via SNPs, which were used as genetic instrumental variables (IVs). The veracity of the causal estimation was ensured through three core assumptions: (I) genetic IVs are strongly associated with HPV-related indicators; (II) genetic IVs are independent of potential confounders; and (III) genetic IVs must influence the occurrence of various types of cancers only through HPV-associated indicators. The directionality of the causal linkage was further determined with a bidirectional Mendelian randomization design, which ruled out a potential reverse causal linkage. A brief description of the study design is shown in Figure 1.

### 2.2. HPV-Related Indicators Exposure Data Source

The HPV16/18 protein exposure dataset was downloaded from access on MRC IEU OpenGWAS (https://gwas.mrcieu.ac.uk/, accessed on 1 August 2023), which was developed at the MRC Integrative Epidemiology Unit at the University of Bristol. The data were derived from a study [19] published in 2017 by Karsten Suhre et al. In this study, Karsten Suhre et al. randomly selected 1000 individuals out of 1800 who had been intensively phenotyped within the KORA F4 [20] cohort. Meanwhile, proteomics platform (SOMAscan) [21] was used to quantify the levels of 1124 proteins from the plasma of the study participants in the GWAS research. HPV16/18 E7 protein was included in the assay of 1124 plasma proteins.

The HPV16 chronic infection dataset was downloaded from GWAS Catalog [22] (https://www.ebi.ac.uk/gwas/, accessed on 5 August 2023). The source of this dataset was a study [23] published in 2019 by Bigyan Mainali et al. The study was conducted on a subcohort of the HPV infection in men (HIM) study, including male patients who were HPV16-positive at least once during the study period, with a predominantly white ethnicity. The control group was defined as men infected with HPV16 who cleared within 18 months and were HPV16-negative at the end of the study, while the case group was defined as HPV16 infections that persisted for 18 months or longer and were still positive at the end of the study. The general information of the above 3 HPV-related indicators exposure datasets were demonstrated in Table S1.

### 2.3. The Selection Criteria of Instrumental Variables for MR Analysis

To ensure the validity of the results in the HPV-related exposure dataset, we used a set of criteria to screen the IVs: (I) IVs were strongly correlated with exposure factors, i.e., *p* < 5 × 10^−5^ for genome-wide significance; (II) to address issues of linkage disequilibrium, we applied a threshold of r^2^ = 0.01 and kb = 5000, removing SNPs with an r^2^ greater than 0.01 within a range of 5000 kb with the most significant SNP; and (III) IVs associated with confounders of the exposure and outcome were removed by checking the secondary phenotype of each SNP on PhenoScanner [24]. The F-statistics were calculated as the following formula [25,26,27]:

$$F=\frac{R^{2}\times \left(N-2\right)}{1-R^{2}}$$

**Figure 2:**
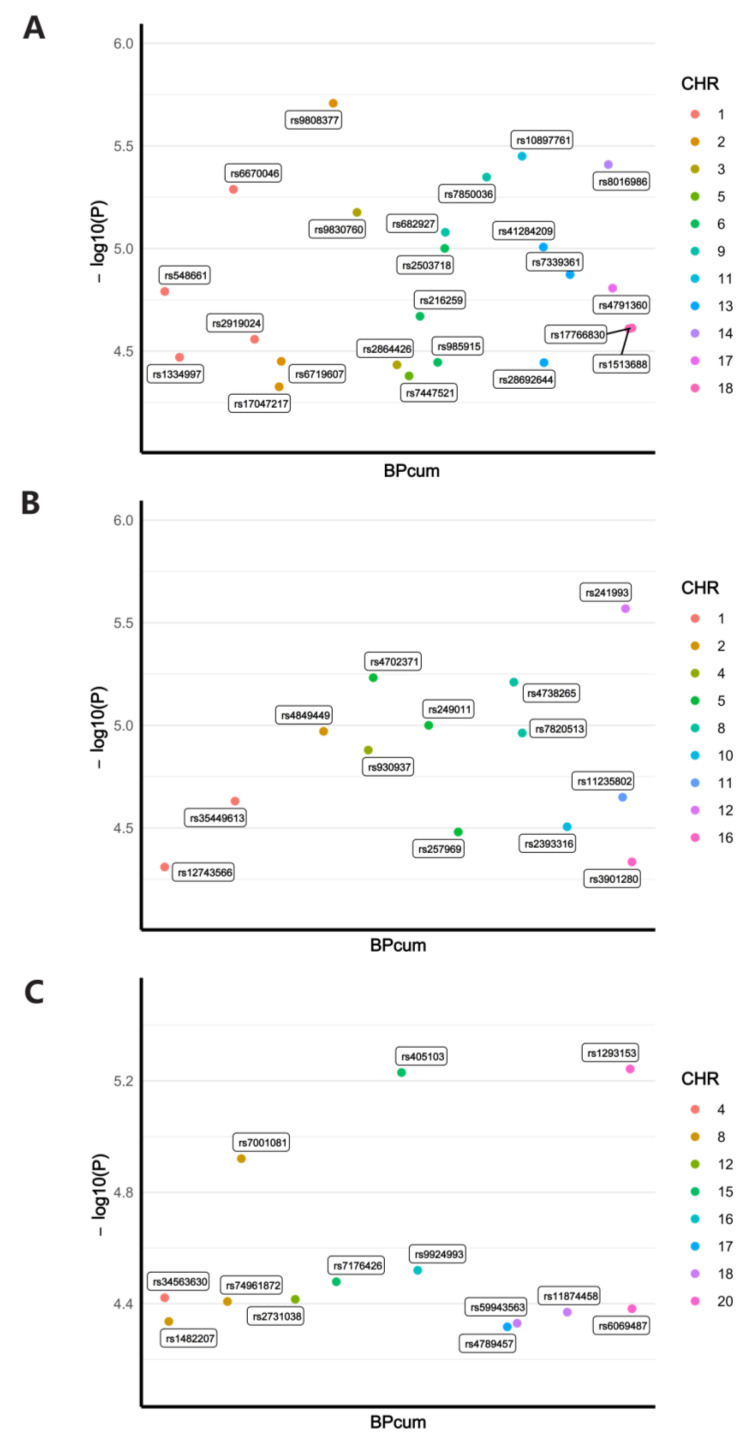
Manhattan plot of the SNPs identified as instrumental variables (IVs) from the three exposure datasets. (**A**) SNPs from the HPV16 E7 protein dataset; (**B**) SNPs from the HPV18 E7 protein dataset; and (**C**) SNPs from the HPV chronic infection dataset.

In this formula, *N* is the sample size in the selected exposure dataset and *R*^2^ represents the degree of variation explained by each SNP, which is calculated as the following formula

$$R^{2}=\frac{2\times \beta^{2}\times EAF\times \left(1-EAF\right)}{2\times \beta^{2}\times EAF\times \left(1-EAF\right)+2\times SE^{2}\times N\times EAF\times \left(1-EAF\right)}$$

where β is the amount of SNP effect on exposure and EAF is the frequency of the effector allele. By calculating the F-statistics, we assessed the correlation strength between IVs and exposure factors, and identified IV as a strong instrumental variable when F > 10. The detailed information of selected SNPs is shown in Figure 2 and Tables S2–S4.

### 2.4. Cancer Outcome Data Source

Through an extensive review of the literature, we conducted a comprehensive screening encompassing 12 cancers and tumors which may have a potential causal relationship with human papillomavirus (HPV), including bladder cancer, anal cancer, prostate cancer, vaginal cancer, breast cancer, colorectal cancer, lung cancer, vulvar cancer, oropharyngeal cancer, head and neck cancer, ovarian cancer, and skin cancer. All cancer-related datasets were downloaded from MRC IEU OpenGWAS. The study population’s ethnicity was predominantly European, consistent with the corresponding exposure dataset. Table S1 provides a comprehensive description of the details of the cancer-related outcome datasets. We ensured that all outcome datasets utilized in the analysis were obtained from distinct research organizations compared to the exposure dataset, thus sample overlap was deemed negligible.

### 2.5. Bidirectional Two-Sample MR Analysis

In this study, 6 different MR methods were utilized to examine the relationship between HPV16/18 protein exposure and cancer outcome. These methods include MR-Egger, weighted median, inverse variance weighted, simple mode, weighted mode, and MR-PRESSO. Our primary focus was directed towards the findings obtained from MR-Egger and inverse variance weighted (IVW) analyses, aiming to evaluate both the statistical significance and the effect of the exposure on the outcome. 

Horizontal pleiotropy was assessed using the *p*-values of MR-Egger’s intercept and MR-PRESSO global test (when *p* > 0.05 is considered to be no horizontal pleiotropy), and a few outliers among the IVs were excluded to guarantee that the IVs exclusively influenced the outcomes through the exposure variable, minimizing the influence of any confounding factors. Additionally, to evaluate the heterogeneity among the IVs, we employed Cochran’s Q-test. Heterogeneity was considered to exist when the Q < 0.05, and the casual effect was re-evaluated with multiple random effects model (MRE). Furthermore, in the leave-one-out test, we conducted MR analyses after removing each IV individually and compared the change of results, removing those whose removal had a greater impact on the overall effect. Ultimately, we conducted a reverse MR analysis to investigate the potential presence of a reverse causal effect through a similar procedure.

### 2.6. MR Analyses in Validation Datasets

To further improve the credibility of the results in the previous MR analyses, we substituted the exposure and outcome datasets and performed validation analyses using two-sample Mendelian randomization. The exposure dataset was replaced with the HPV16 chronic infection dataset from the GWAS Catalog, and the outcome dataset was replaced with other cancer datasets from OpenGwas. The datasets used for validation are shown in Table S1.

### 2.7. Immune Infiltration Analysis in HPV Associated Cancers

HPVTIMER is a web-based online analysis platform that integrates the expression data from Gene Expression Omnibus (GEO), including 8 HPV-associated cancers, 65 transcriptomic datasets, 2290 samples, and more than 10,000 genes [28]. Using HPVTIMER, we performed immune infiltration analyses in head and neck squamous cell carcinoma (HNSCC), oropharyngeal squamous cell carcinoma (OPSCC) and vulval squamous cell carcinoma (VSCC) to evaluate the tumor immune microenvironment of these tumors. CIBERSORT algorithm was adopted to investigate the differences in immune cell scores between HPV-positive and HPV-negative groups. *p* < 0.001 was considered a statistically significant difference in the immune infiltration analysis.

### 2.8. Statistical Analyses

All statistical analyses were performed in R software (version 4.2.2). R packages utilized for data collation, MR analysis, charting include “TwoSampleMR”, “friendly2MR”, “qqman”, “dplyr”, “tidyverse”, “patchwork”, “ggpubr”, “grid”, and “forestploter”. A bilateral *p* < 0.05 was considered statistically significant.

## 3. Results

### 3.1. Detailed Information of Screened IVs

Based on the previously described criteria, we screened SNPs from the three exposure datasets. A total of 23 SNPs were obtained from the HPV16 E7 exposure dataset, 13 SNPs from the HPV18 E7 exposure dataset, and 13 SNPs from the HPV16 chronic infection dataset. The detailed information of these datasets is displayed in Supplementary Tables S2–S4. The Manhattan plot illustrates the *p*-value of each SNP and its relative position on the chromosome (Figure 2). The F-statistics of the IVs obtained from the three exposure datasets were 18.97, 18.90, and 17.43, respectively. No F-statistics were less than 10, which indicates that there existed no bias due to weak instrumental variables, and the assumption I of MR is satisfied. Based on PhenoScanner, we examined the obtained SNPs and found that these SNPs were not significantly correlated (*p* < 5 × 10^−5^) with any of the 12 cancers of our interest and their associated confounders. In subsequent MR analyses, these SNPs were used as IVs to investigate causal effects between HPV infection and cancers.

### 3.2. Causal Effect between Plasma Levels of HPV E7 Protein and Cancers

**Figure 3:**
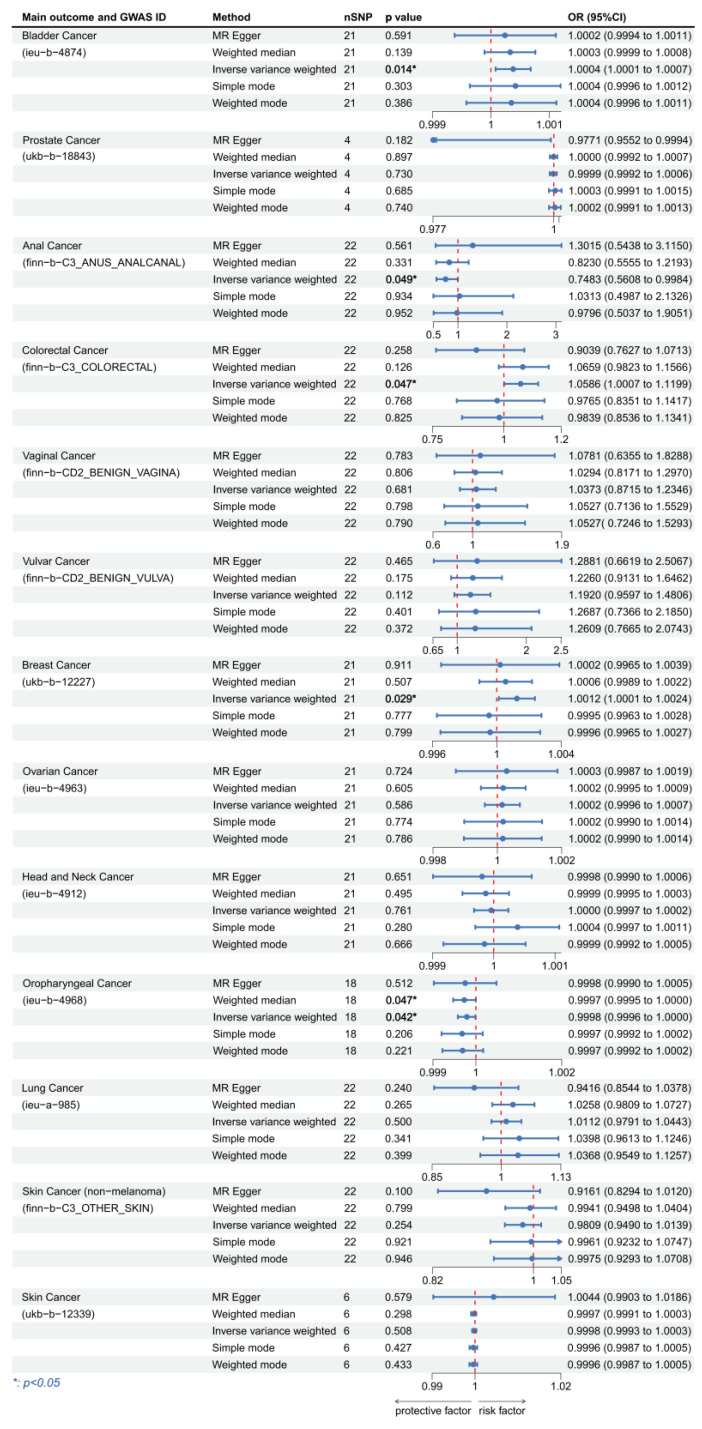
Forest plot of two-sample Mendelian randomization (MR) estimation of the association between HPV16 E7 protein and cancer risk. Note: nSNP, number of single nucleotide polymorphisms; CI, confidence interval.

**Figure 4:**
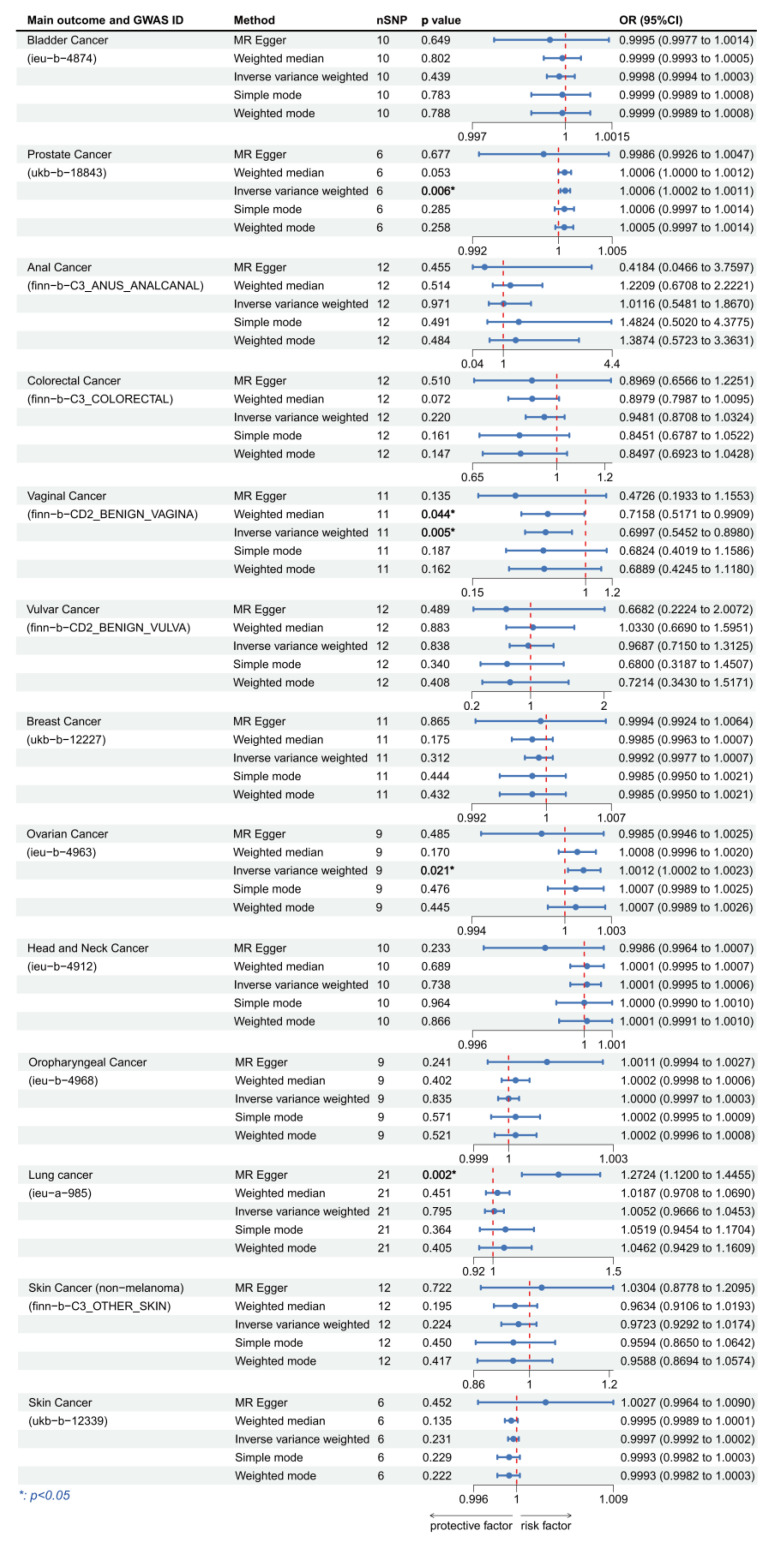
Forest plot of two-sample Mendelian randomization (MR) estimation of the association between HPV18 E7 protein and cancer risk. Note: nSNP, number of single nucleotide polymorphisms; CI, confidence interval.

We focused on the results of the MR-Egger method as well as the IVW method, and all the results are presented in Figure 3 and Figure 4. When using HPV16 E7 protein as the exposure, the linkages were significant in bladder cancer, anal cancer, colorectal cancer, breast cancer, and oropharyngeal cancer. We discovered that HPV16 E7 protein exposure was a risk factor for bladder cancer (IVW: OR per unit increase in protein level = 1.000377, 95% CI: 1.000077–1.000677, *p* = 0.014), colorectal cancer (IVW: OR per unit increase in protein level = 1.058581, 95% CI: 1.000655–1.119861, *p* = 0.047) and breast cancer (IVW: OR per unit increase in protein level = 1.001249, 95% CI: 1.000127–1.002373, *p* = 0.029), and a protective factor for anal cancer (IVW: OR per unit increase in protein level = 0.748302, 95% CI: 0.560843–0.998418, *p* = 0.049) and oropharyngeal cancer (IVW: OR per unit increase in protein level = 0.999786, 95% CI: 0.99958–0.999992, *p* = 0.042). When considering HPV18 E7 protein as the exposure, we found significant linkage in prostate cancer, vaginal cancer, ovarian cancer, and lung cancer. HPV18 E7 protein exposure was a risk factor for prostate cancer (IVW: OR per unit increase in protein level = 1.000634, 95% CI: 1.000178–1.001090, *p* = 0.006), ovarian cancer (IVW: OR per unit increase in protein level = 1.001249, 95% CI: 1.000191–1.002309, *p* = 0.021), and lung cancer (MR-Egger: OR per unit increase in protein level = 1.261943, 95% CI: 1.067232–1.492177, *p* = 0.022), and was a protective factor for vaginal cancer (IVW: OR per unit increase in protein level = 0.699719, 95% CI: 0.545211–0.898014, *p* = 0.005). No statistically significant results were achieved in any of the other cancers.

Scatter plot further visualized the effect of IVs on exposure and outcome. A positive slope in the scatter plot suggests an inverse correlation between the exposure and the outcome, and vice versa. Supplementary Figures S1 and S2 displayed the respective scatter plots for the two exposure datasets. Additionally, Supplementary Figures S3 and S4 depicted funnel plots employed to evaluate any potential bias from IVs. However, the number of IVs used in this study was not enough, making it difficult to visually assess the symmetry of the funnel plots. The effects of individual SNPs in each set of MR analyses and their combined effects are shown in the forest plots (Supplementary Figures S5 and S6).

We also performed inverse MR analyses for all of the above exposures and outcomes to eliminate reverse causality, and the results are shown in Supplementary Table S5. The reverse MR analyses for prostate and skin cancers did not produce valid results due to insufficient number of IVs, while the remaining reverse MR analyses did not show significant results, suggesting that there existed no potential reverse causality.

### 3.3. Validation of MR Analysis Results

**Figure 5:**
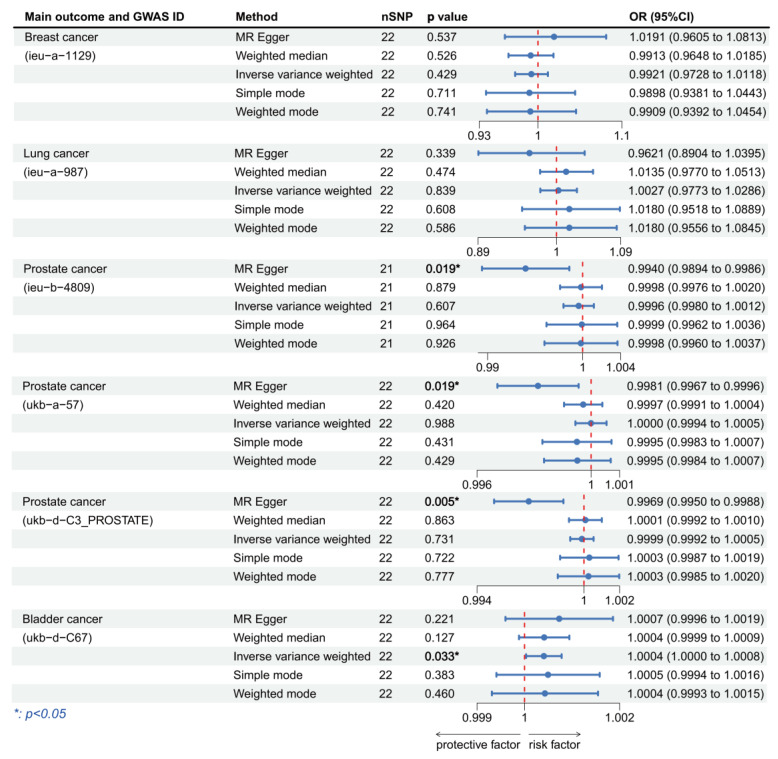
Forest plot of two-sample Mendelian randomization (MR) estimation of the association between HPV16 E7 protein and cancer risk for validation analysis. Note: nSNP, number of single nucleotide polymorphisms; CI, confidence interval.

**Figure 6:**
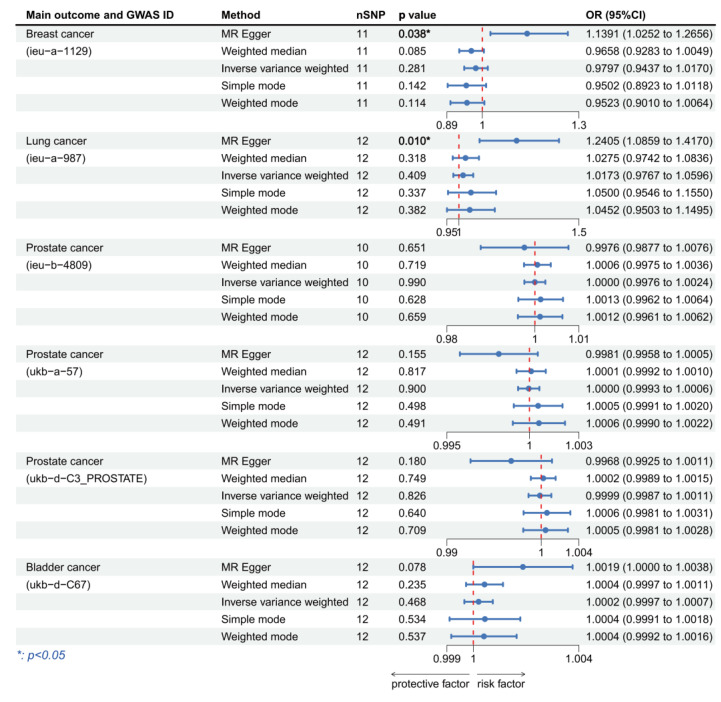
Forest plot of two-sample Mendelian randomization (MR) estimation of the association between HPV18 E7 protein and cancer risk for validation analysis. Note: nSNP, number of single nucleotide polymorphisms; CI, confidence interval.

**Figure 7:**
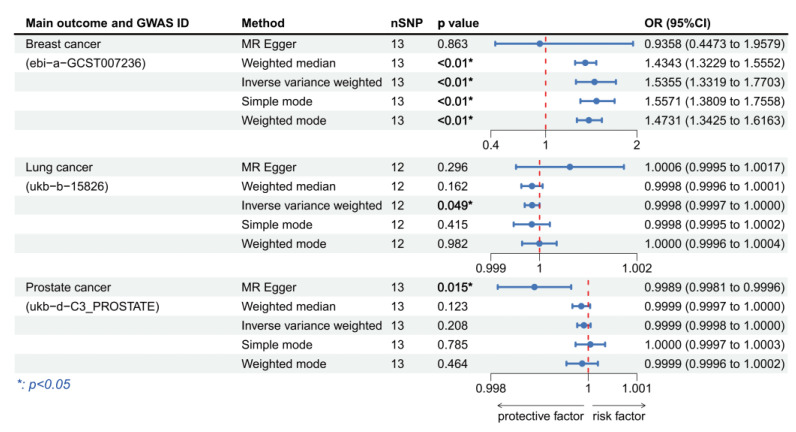
Forest plot of two-sample Mendelian randomization (MR) estimation of the association between HPV chronic infection and cancer risk for validation analysis. Note: nSNP, number of single nucleotide polymorphisms; CI, confidence interval.

To further improve the reliability of the conclusions of the previous MR analyses, we replaced the outcome and exposure datasets in the cancers of interest and repeated the MR analyses. In the MR analysis of HPV16 E7 protein exposure, we obtained significance results in three prostate cancer datasets and one bladder cancer dataset (Figure 5). All the three prostate cancer datasets were significant using the MR-Egger method and all showed HPV16 E7 protein exposure was a protective factor (OR per unit increase in protein level = 0.993995, 95% CI: 0.989414–0.998597, *p* = 0.019; OR per unit increase in protein level = 0.998138, 95% CI: 0.996714–0.999563, *p* = 0.019; OR per unit increase in protein level= 0.996901, 95% CI: 0.994958–0.998847, *p* = 0.005). The bladder cancer dataset results showed HPV16 E7 protein exposure was a significant risk factor (IVW: OR per unit increase in protein level = 1.000406, 95% CI: 1.000033–1.000780, *p* = 0.033). In the MR analysis of HPV18 E7 protein exposure, significant results were found in breast cancer and lung cancer. We discovered that HPV18 E7 protein exposure was a risk factor for both breast cancer (MR-Egger: OR per unit increase in protein level = 1.139096, 95% CI: 1.025223–1.265617, *p* = 0.038) and lung cancer (MR-Egger: OR per unit increase in protein level = 1.261943, 95% CI: 1.067232–1.492177, *p* = 0.022) (Figure 6). Although no significant results were obtained in the prostate cancer dataset, most of the ORs in this MR analysis were >1, which is consistent with the previous analyses. In addition, we used HPV16 chronic infection dataset as the exposure dataset for further validation and obtained significant results in breast cancer, lung cancer and prostate cancer. Chronic HPV16 infection appears to be a significant risk factor for breast cancer (IVW: OR = 1.535548, 95% CI: 1.331936–1.770286, *p* = 3.436 × 10^−9^) and a significant protective factor for lung cancer (IVW: OR = 0.999847, 95% CI: 0.999694–1.000000, *p* = 0.049) and prostate cancer (IVW: OR = 0.998894, 95% CI: 0.998140–0.999650, *p* = 0.015) (Figure 7). Supplementary Figures S7–S9 displayed the respective scatter plots for these three validation analyses. Funnel plots illustrated the possible bias in validation analyses (Supplementary Figures S10–S12). However, due to the insufficient number of independent variables, direct assessment was still difficult. The effects of individual SNPs in each set of MR analyses and their combined effects are shown in the forest plots (Supplementary Figures S13–S15).

### 3.4. Sensitivity Analysis of the MR Results

Sensitivity analyses were conducted to further assess the robustness of the results as well as the potential bias in both primary and validation MR analyses. MR-Egger regression analysis and the MR-PRESSO global test were both utilized for horizontal pleiotropy assessment (Table 1, Table 2, Table 3, Table 4 and Table 5). In MR analyses with HPV16 E7 as the exposure, the intercept terms of the MR-Egger results for three prostate cancer datasets were significant (Table 3); in MR analyses with HPV18 E7 as the exposure, the intercept terms of the MR-Egger results for two lung cancer datasets and a breast cancer dataset were significant (Table 2 and Table 4), and the MR-PRESSO global test *p* value of anal cancer <0.05 (Table 2); in MR analyses with HPV16 chronic infection as the exposure, the intercept terms of the MR-Egger results for the prostate cancer dataset was significant and the MR-PRESSO global test *p* value of breast cancer <0.05 (Table 5). Apart from the above results, the MR-Egger regression analysis and MR-PRESSO global test results of the remaining MR analyses were insignificant, indicating the absence of horizontal pleiotropy.

The leave-one-out test demonstrates the causal effect of eliminating each individual SNP compared to the IVW method (Supplementary Figures S16–S20). The results revealed that a general consensus is maintained between the two methods. Cochran’s Q-test was employed to evaluate the heterogeneity of each MR analysis and the results are shown in Table 1, Table 2, Table 3, Table 4 and Table 5. Significant heterogeneity was identified in the MR analyses that considered HPV18 E7 as the exposure and anal cancer as the outcome, and HPV chronic infection as the exposure and breast cancer as the outcome (Table 2 and Table 5). Outcomes with heterogeneity were reevaluated using multiple random effects models, and the results re-estimated for both anal cancer (IVWMRE: OR = 1.011564, 95% CI: 0.5480772–1.867002, *p* = 0.970) and breast cancer (IVWMRE: OR = 1.535548, 95% CI: 1.331936–1.770286, *p* = 3.43 × 10^−9^) were consistent with the MR results, indicating that the causal effect remains valid. All other MR analyses revealed no significant heterogeneity in their results.

### 3.5. Immune Infiltration Analysis

**Figure 8:**
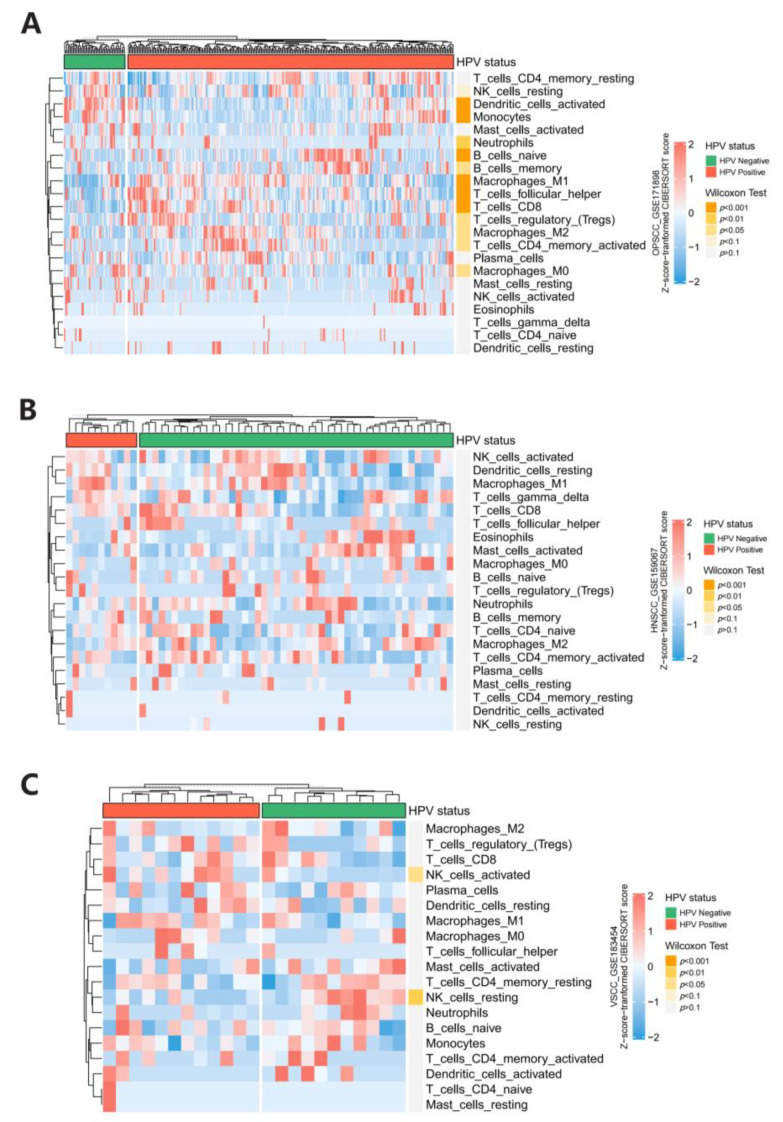
Heatmap of immune infiltration analysis for (**A**) oropharyngeal squamous cell carcinoma (OPSCC), (**B**) head and neck squamous cell carcinoma (HNSCC), and (**C**) vulval squamous cell carcinoma (VSCC).

In order to explore the immunomodulatory effect of HPV infection, we then investigated the immune infiltration status in the tumor microenvironment using HPVTIMER. As for OPSCC, we found that, compared to patients in the HPV-negative group, those in the HPV-positive group had significantly more immune cell infiltration in the tumor microenvironment, especially naive B cells, M1 Macrophages, follicular helper T cells, and CD8+ T cells (*p* < 0.001), which primarily exerted anti-tumor effects and might contribute to the protective effect of HPV infection for tumorigenesis to some extent (Figure 8A). In addition, there were no significant differences in the immune infiltration in the tumor environment of HNSCC and VSCC (Figure 8B,C).

## 4. Discussion

In 1976, Hausen first proposed a hypothesis that the development of cervical cancer correlated with HPV infection [29], which resulted in decades of research on HPVs. To date, more and more new types of HPVs have been identified. The relationship between HPV and cervical cancer has been thoroughly researched, and unambiguous conclusions have been reached. HPV16 and HPV18, two typical high-risk oncological HPVs, both belong to Alphapapillomaviruses, and their oncological effects have been clearly demonstrated. After entering the nucleus by endosomes, HPVs intergrade to the DNA of hosts and dysregulate the expression level of many proteins, especially oncoproteins E6 and E7. E6 and E7 proteins derive from the viral genome, and are the key proteins moderating the cellular environment for viral replication. E7 proteins from all genera can bind to Rb protein, but only oncological E7 protein can degrade Rb protein, thus inducing p53 [30]. Similarly, E6 proteins from all Alphapapillomaviruses can form a complex with E3 ligase E6 associated protein (E6AP), and disrupt the transactivation of p53 [31]. However, only oncological E6 protein can degrade p53 with E3 ubiquitin ligase function from E6AP. In the synergies of E7 and E6 protein, cells would not undergo growth arrest and finally, this leads to oncogenesis. In the human body, research showed that the HPV E7 protein was a novel and specific test that can be used to differentiate transient HPV infection and malignant or pre-malignant lesions [32].

HPVs from different genera have different infection cycles in human hosts. Although the course of infection depends on the balance between HPV infection and host immunity to a large extent, most HPVs tend to cause chronic infections. HPVs from Betapapillomavirus and Gammapapillomavirus usually cause asymptomatic infection, and the infection might persist for many years. On the other hand, before the development of cervical cancer, the oncological HPV-induced lesions could exist for more than 10 years. Moreover, the latest evidence indicates that HPVs might cause latent infections [33], and this characteristic probably correlates with a second peak of cervical infection with the same HPV type in some older women [10]. All these results highlight the importance of early prevention and early intervention. Exhilaratingly, the utility of HPV vaccination significantly decreases the incidence of cervical cancer, which is a key step for humans to overcome HPV-associated cancers. Since 2006, three successful HPV vaccinations have been used worldwide, and have eliminated approximately 90% of HPV-induced cervical cancer and 70% of anogenital warts [11]. Next, apart from popularizing the HPV vaccination and developing new vaccinations with higher potency, discovering the causal effects between HPVs and other cancers, and assessing the protective effects of HPV vaccination on other cancers are also particularly important. In this MR analysis, we aimed to reveal the causal relationship between hrHPV (HPV16 and HPV18) infection and pan-cancer development.

Lung cancer is one of the most commonly diagnosed cancers worldwide. The incidence of lung cancer in non-smokers has been comprehensively studied for many years. Previous studies revealed that several types of HPVs have been found in the lung tissue of lung cancer patients [34], and the infection of HPV16/18 in lung tissue was associated with the development of lung cancer [35]. In this study, we further confirmed this correlation that HPV18 E7 protein exposure was associated with a higher risk of the development of lung cancer. Nevertheless, we found that HPV16 chronic infection was a protective factor for lung cancer. However, this result was only obtained in validation MR analyses with chronic HPV infection as the exposure dataset, and should therefore be treated with caution.

Due to the anatomical relevance of the urinary tract and genitalia, the relationship between HPV infection and urological tumor also attracts great interest from researchers. Previous studies presented conflicting results about the association between HPV infection and the development of bladder cancer [36,37]. Here, we found that HPV16 was associated with a higher risk of bladder cancer in both original datasets and verification datasets. As for prostate cancer, the majority view is that HPV16 and HPV18 infections are not associated with prostate cancer [38,39]. However, our MR analysis revealed that HPV16 infection had a protective effect on prostate cancer, while HPV18 infection was a risk factor for prostate cancer. In particular, that HPV16 is protective against prostate cancer was validated in three different outcome datasets (Figure 5). Since Mendelian randomization has a higher evidence level than retrospective studies, this result may suggest that HPV infection is causally associated with prostate cancer, but further studies are needed to explore the underlying mechanisms.

Since higher HPV viral loads were discovered in some anal patients, it seems that HPV infection is associated with a higher risk of anal cancer. However, previous studies also showed that anal cancer development was not associated with HPV16 infection [40], and higher HPV16 viral loads were associated with a better anal cancer prognosis after adjusting for other clinical factors [41]. Interestingly, in this MR analysis, we found that HPV16 was a protective factor for anal cancer, which brought a new insight about HPV infection and cancer development. This protective effect might be partially ascribed to the activation of immunity by HPV16 infection. Previous systematic reviews found a higher prevalence of HPV16/18 DNA in colorectal cancer populations compared with healthy controls [42], suggesting an association between HPV and the development of colorectal cancer, but no conclusions on causality were summarized. The results of our MR analysis showed that HPV16 was a risk factor for colorectal cancer, which further demonstrated a causal link between them.

HPV is now mainly considered a cofactor or mediator of breast cancer [43], since there still lacks key evidence about the casual link between them. Our MR analysis suggests that HPV16/18 is a risk factor for breast cancer from a causal perspective. As for vaginal cancer, a Chinese population-based study showed that HPV was associated with about 70% of vaginal cancers [44]. However, in the aspect of clinical practice, another study showed that patients with HPV-positive vaginal cancer had a better prognosis than HPV-negative patients [45]. Here, our MR results suggested that HPV18 is a protective factor against vaginal cancer. Based on these results, we might need to examine the casual relationship between HPV and vaginal cancer again. We hypothesize that the immune-activating effect of HPV provides protection against vaginal cancer.

The current mainstream view generally recognizes a facilitating role of HPV infection in oropharyngeal tumorigenesis [46], but there are also many studies showing that patients with HPV-positive oropharyngeal cancer are more susceptible to radiotherapy and chemotherapy and have a better overall prognosis [47,48,49]. The MR results showed that HPV E7 protein exposure was a protective factor against oropharyngeal cancer. Mendelian randomization is theoretically of a higher level of evidence compared with retrospective studies. In addition, some studies have shown that HPV16-positive oropharyngeal cancer patients have higher levels of lymphocytes infiltration in the tumor microenvironment [50], while our immune infiltration analysis also showed that HPV-positive patients have higher levels of tumor-killing immune cells. This may suggest that HPV has some kind of protective effect against oropharyngeal cancer by activating immunity and recruiting immune cells. In general, our MR provided a new insight about HPV infection and cancer development. For anal, vaginal and oropharyngeal cancer, the casual link between HPV and cancer should be re-evaluated, and the balance between oncological effects of HPV and immune activation should be carefully investigated.

However, our research was subject to some objective limitations. Several databases were searched, including IEU Open GWAS, GWAS Catalog, UKBiobank, and GWAS Atlas, etc. However, only three HPV-related datasets in this study were available, and these three exposure datasets have small sample sizes, potentially rendering them unrepresentative. Secondly, for the purpose of acquiring a satisfactory quantity of IVs, we applied relatively broad SNP screening criteria. Although the criteria can ensure the validity of the correlation hypothesis, it may not entail a very strong association between IVs and exposure. Moreover, the study population in this research was mainly of European origin and may not be necessarily generalizable to other ethnicities. Finally, MR analyses can only provide valid inferences of causal effects, but cannot reveal the intrinsic mechanisms between HPV and cancer development, which require further exploration. The opinion that HPV infection has a protective effect against cancer requires further experimental and clinical trials for validation.

## 5. Conclusions

This study investigated the causal relationship between HPV and several types of cancer using MR analysis. HPV16 was identified as a risk factor for bladder cancer, colorectal cancer and breast cancer, while HPV18 was identified as a risk factor for prostate cancer, ovarian cancer, lung cancer and breast cancer. In these cancers, it may be the predominant oncogenic effect of HPV infection that promotes cancer development. Interestingly, we found that HPV16 may be a protective factor for prostate cancer, anal cancer and lung cancer, and HPV18 may be a protective factor for vaginal cancer. In these cancers, immune activation and local immune cell recruitment by HPV infection may predominate. These findings provide new insights compared to the prevailing view that HPV infection increases the risk of cancers. Although there may be some limitations to these findings, it suggests that more in-depth mechanistic studies, as well as epidemiological investigations, are required to explore the role of HPV in the development of these cancers.

## Figures and Tables

**Table 1:** The sensitivity analysis of the results of HPV16 E7 protein-cancer MR analysis.

Main Outcome	Method	Cochran’s Q-Test.p	MR.Egger.Intercept.p	MR.PRESSO.Global.Test.p
Bladder Cancer	MR-Egger	0.55	0.747	
	Inverse variance weighted	0.61		0.647 (raw, 0 outliers)
Prostate Cancer	MR-Egger	0.97	0.184	
	Inverse variance weighted	0.25		0.352 (raw, 0 outliers)
Anal Cancer	MR-Egger	0.50	0.203	
	Inverse variance weighted	0.46		0.504 (raw, 0 outliers)
Colorectal Cancer	MR-Egger	0.74	0.068	
	Inverse variance weighted	0.57		0.594 (raw, 0 outliers)
Vaginal Cancer	MR-Egger	0.75	0.881	
	Inverse variance weighted	0.80		0.820 (raw, 0 outliers)
Vulvar Cancer	MR-Egger	0.43	0.811	
	Inverse variance weighted	0.48		0.529 (raw, 0 outliers)
Breast Cancer	MR-Egger	0.35	0.567	
	Inverse variance weighted	0.38		0.421 (raw, 0 outliers)
Ovarian Cancer	MR-Egger	1.00	0.858	
	Inverse variance weighted	1.00		0.998 (raw, 0 outliers)
Head and Neck Cancer	MR-Egger	0.93	0.710	
	Inverse variance weighted	0.95		0.955 (raw, 0 outliers)
Oropharyngeal Cancer	MR-Egger	0.97	0.922	
	Inverse variance weighted	0.98		0.978 (raw, 0 outliers)
Lung Cancer	MR-Egger	0.86	0.144	
	Inverse variance weighted	0.78		0.771 (raw, 0 outliers)
Skin Cancer (non-melanoma)	MR-Egger	0.50	0.169	
	Inverse variance weighted	0.44		0.481 (raw, 0 outliers)
Skin Cancer	MR-Egger	0.85	0.565	
	Inverse variance weighted	0.88		0.877 (raw, 0 outliers)

**Table 2:** The sensitivity analysis of the results of HPV18 E7 protein-cancer MR analysis. (*: *p* < 0.05).

Main Outcome	Method	Cochran’s Q-Test.p	MR.Egger.Intercept.p	MR.PRESSO.Global.Test.p
Bladder Cancer	MR-Egger	0.66	0.775	
	Inverse variance weighted	0.74		0.748 (raw, 0 outliers)
Prostate Cancer	MR-Egger	0.39	0.548	
	Inverse variance weighted	0.48		0.53 (raw, 0 outliers)
Anal Cancer	MR-Egger	0.01 *	0.430	
	Inverse variance weighted	0.01 *		0.011 (outlier corrected, 1 outlier) *
Colorectal Cancer	MR-Egger	0.22	0.723	
	Inverse variance weighted	0.28		0.289 (raw, 0 outliers)
Vaginal Cancer	MR-Egger	0.97	0.394	
	Inverse variance weighted	0.97		0.82 (raw, 0 outliers)
Vulvar Cancer	MR-Egger	0.37	0.506	
	Inverse variance weighted	0.41		0.424 (raw, 0 outliers)
Breast Cancer	MR-Egger	0.37	0.963	
	Inverse variance weighted	0.46		0.502 (raw, 0 outliers)
Ovarian Cancer	MR-Egger	0.23	0.202	
	Inverse variance weighted	0.15		0.175 (raw, 0 outliers)
Head and Neck Cancer	MR-Egger	0.13	0.194	
	Inverse variance weighted	0.08		0.082 (raw, 0 outliers)
Oropharyngeal Cancer	MR-Egger	0.47	0.247	
	Inverse variance weighted	0.42		0.439 (raw, 0 outliers)
Lung Cancer	MR-Egger	0.79	0.019*	
	Inverse variance weighted	0.23		0.233 (raw, 0 outliers)
Skin Cancer (non-melanoma)	MR-Egger	0.90	0.476	
	Inverse variance weighted	0.91		0.909 (raw, 0 outliers)
Skin Cancer	MR-Egger	0.48	0.407	
	Inverse variance weighted	0.50		0.532 (raw, 0 outliers)

**Table 3:** The sensitivity analysis of the results of HPV16 E7 protein-cancer validation MR analysis. (*: *p* < 0.05).

Main Outcome	Method	Cochran’s Q-Test.p	MR.Egger.Intercept.p	MR.PRESSO.Global.Test.p
Breast cancer	MR-Egger	0.53	0.357	0.541 (raw, 0 outliers)
(ieu-a-1129)	Inverse variance weighted	0.54		
Lung cancer	MR-Egger	0.91	0.122	0.860 (raw, 0 outliers)
(ieu-a-987)	Inverse variance weighted	0.86		
Prostate cancer	MR-Egger	0.89	0.020 *	0.543 (raw, 0 outliers)
(ieu-b-4809)	Inverse variance weighted	0.57		
Prostate cancer	MR-Egger	0.31	0.013 *	0.076 (raw, 0 outliers)
(ukb-a-57)	Inverse variance weighted	0.08		
Prostate cancer	MR-Egger	0.97	0.005 *	0.508 (raw, 0 outliers)
(ukb-d-C3_PROSTATE)	Inverse variance weighted	0.54		
Bladder cancer	MR-Egger	0.32	0.560	0.370 (raw, 0 outliers)
(ukb-d-C67)	Inverse variance weighted	0.35		

**Table 4:** The sensitivity analysis of the results of HPV18 E7 protein-cancer validation MR analysis. (*: *p* < 0.05).

Main Outcome	Method	Cochran’s Q-Test.p	MR.Egger.Intercept.p	MR.PRESSO.Global.Test.p
Breast cancer	MR-Egger	0.53	0.017 *	0.541 (raw, 0 outliers)
(ieu-a-1129)	Inverse variance weighted	0.09		
Lung cancer	MR-Egger	0.96	0.001 *	0.134 (raw, 0 outliers)
(ieu-a-987)	Inverse variance weighted	0.14		
Prostate cancer	MR-Egger	0.71	0.643	0.790 (raw, 0 outliers)
(ieu-b-4809)	Inverse variance weighted	0.77		
Prostate cancer	MR-Egger	0.52	0.149	0.400 (raw, 0 outliers)
(ukb-a-57)	Inverse variance weighted	0.40		
Prostate cancer	MR-Egger	0.10	0.182	0.072 (raw, 0 outliers)
(ukb-d-C3_PROSTATE)	Inverse variance weighted	0.06		
Bladder cancer	MR-Egger	0.49	0.099	0.314 (raw, 0 outliers)
(ukb-d-C67)	Inverse variance weighted	0.31		

**Table 5:** The sensitivity analysis of the results of HPV chronic infection-cancer validation MR analysis. (*: *p* < 0.05).

Main Outcome	Method	Cochran’s Q-Test.p	MR.Egger.Intercept.p	MR.PRESSO.Global.Test.p
Breast cancer	MR-Egger	<0.01 *	0.208	<0.001 (outlier corrected, 1 outlier) *
(ebi-a-GCST007236)	Inverse variance weighted	<0.01 *		
Lung cancer	MR-Egger	0.52	0.196	0.460 (raw, 0 outliers)
(ukb-b-15826)	Inverse variance weighted	0.44		
Prostate cancer	MR-Egger	0.81	0.022*	0.312 (raw, 0 outliers)
(ukb-d-C3_PROSTATE)	Inverse variance weighted	0.30		

## Data Availability

The data presented in this study are openly available in OpenGWAS and GWAS Catalog.

## Supplementary Materials

The following supporting information can be downloaded at: https://www.mdpi.com/article/10.3390/cancers15215147/s1, Figure S1: Scatter plots of SNPs associated with HPV 16 E7 protein and risk on (A) bladder cancer, (B) prostate cancer, (C) anal cancer, (D) colorectal cancer, (E) vaginal cancer, (F) vulvar cancer, (G) breast cancer, (H) ovarian cancer, (I) head and neck cancer, (J) oropharyngeal cancer, (K) lung cancer, (L) skin cancer (non−melanoma), (M) skin cancer incidence; Figure S2: Scatter plots of SNPs associated with HPV 18 E7 protein and risk on (A) bladder cancer, (B) prostate cancer, (C) anal cancer, (D) colorectal cancer, (E) vaginal cancer, (F) vulvar cancer, (G) breast cancer, (H) ovarian cancer, (I) head and neck cancer, (J) oropharyngeal cancer, (K) lung cancer, (L) skin cancer (non−melanoma), (M) skin cancer incidence; Figure S3: Funnel plots of the relationship between the causal effect of HPV 16 E7 protein and site-specific cancers. (A) bladder cancer, (B) prostate cancer, (C) anal cancer, (D) colorectal cancer, (E) vaginal cancer, (F) vulvar cancer, (G) breast cancer, (H) ovarian cancer, (I) head and neck cancer, (J) oropharyngeal cancer, (K) lung cancer, (L) skin cancer (non−melanoma), (M) skin cancer incidence; Figure S4: Funnel plots of the relationship between the causal effect of HPV 18 E7 protein and site-specific cancers. (A) bladder cancer, (B) prostate cancer, (C) anal cancer, (D) colorectal cancer, (E) vaginal cancer, (F) vulvar cancer, (G) breast cancer, (H) ovarian cancer, (I) head and neck cancer, (J) oropharyngeal cancer, (K) lung cancer, (L) skin cancer (non−melanoma), (M) skin cancer incidence; Figure S5: Forest plots of each SNP and total effect of HPV 16 E7 protein on (A) bladder cancer, (B) prostate cancer, (C) anal cancer, (D) colorectal cancer, (E) vaginal cancer, (F) vulvar cancer, (G) breast cancer, (H) ovarian cancer, (I) head and neck cancer, (J) oropharyngeal cancer, (K) lung cancer, (L) skin cancer (non−melanoma), (M) skin cancer incidence; Figure S6: Forest plots of each SNP and total effect of HPV 18 E7 protein on (A) bladder cancer, (B) prostate cancer, (C) anal cancer, (D) colorectal cancer, (E) vaginal cancer, (F) vulvar cancer, (G) breast cancer, (H) ovarian cancer, (I) head and neck cancer, (J) oropharyngeal cancer, (K) lung cancer, (L) skin cancer (non−melanoma), (M) skin cancer incidence; Figure S7: Scatter plots of SNPs associated with HPV 16 E7 protein and risk on (A) breast cancer, (B) lung cancer, (C–E) prostate cancer, (F) bladder cancer for validation; Figure S8: Scatter plots of SNPs associated with HPV 18 E7 protein and risk on (A) breast cancer, (B) lung cancer, (C–E) prostate cancer, (F) bladder cancer for validation; Figure S9: Scatter plots of SNPs associated with HPV chronic infection and risk on (A) breast cancer, (B) lung cancer, (C) prostate cancer for validation; Figure S10: Funnel plots of the relationship between the causal effect of HPV 16 E7 protein and site-specific cancers for validation. (A) breast cancer, (B) lung cancer, (C–E) prostate cancer, (F) bladder cancer; Figure S11: Funnel plots of the relationship between the causal effect of HPV 18 E7 protein and site-specific cancers for validation. (A) breast cancer, (B) lung cancer, (C–E) prostate cancer, (F) bladder cancer; Figure S12: Funnel plots of the relationship between the causal effect of HPV chronic infection and site-specific cancers for validation. (A) breast cancer, (B) lung cancer, (C) prostate cancer for validation; Figure S13: Forest plots of each SNP and total effect of HPV 16 E7 protein on (A) breast cancer, (B) lung cancer, (C–E) prostate cancer, (F) bladder cancer for validation; Figure S14: Forest plots of each SNP and total effect of HPV 18 E7 protein on (A) breast cancer, (B) lung cancer, (C–E) prostate cancer, (F) bladder cancer for validation; Figure S15: Forest plots of each SNP and total effect of HPV chronic infection on (A) breast cancer, (B) lung cancer, (C) prostate cancer for validation; Figure S16: Leave-one-out analysis results of SNPs associated with HPV 16 E7 protein and risk on (A) bladder cancer, (B) prostate cancer, (C) anal cancer, (D) colorectal cancer, (E) vaginal cancer, (F) vulvar cancer, (G) breast cancer, (H) ovarian cancer, (I) head and neck cancer, (J) oropharyngeal cancer, (K) lung cancer, (L) skin cancer (non−melanoma), (M) skin cancer incidence; Figure S17: Leave-one-out analysis results of SNPs associated with HPV 18 E7 protein and risk on (A) bladder cancer, (B) prostate cancer, (C) anal cancer, (D) colorectal cancer, (E) vaginal cancer, (F) vulvar cancer, (G) breast cancer, (H) ovarian cancer, (I) head and neck cancer, (J) oropharyngeal cancer, (K) lung cancer, (L) skin cancer (non−melanoma), (M) skin cancer incidence; Figure S18: Leave-one-out analysis results of SNPs associated with HPV 16 E7 protein and risk on (A) breast cancer, (B) lung cancer, (C–E) prostate cancer, (F) bladder cancer incidence for validation; Figure S19: Leave-one-out analysis results of SNPs associated with HPV 18 E7 protein and risk on (A) breast cancer, (B) lung cance, (C–E) prostate cancer, (F) bladder cancer incidence for validation; Figure S20: Leave-one-out analysis results of SNPs associated with HPV chronic infection and risk on (A) breast cancer, (B) lung cancer, (C) prostate cancer incidence for validation; Table S1: The detailed information of all statistical summary datasets used in this study; Table S2: The information of all SNPs from HPV16 E7 protein dataset; Table S3: The information of all SNPs from HPV18 E7 protein dataset; Table S4: The information of all SNPs from HPV chronic infection dataset; Table S5: The results of reverse MR analysis.
