# Supplementary material for: Unveiling the Association between HPV and Pan-Cancers: A Bidirectional Two-Sample Mendelian Randomization Study
Source: Cancers (Basel). 2023 Oct 26;15(21):5147. doi: 10.3390/cancers15215147 (PMC10650873; doi:10.3390/cancers15215147)
Supplement: Supplementary file 1 [file cancers-15-05147-s001.zip › Supplementary Figures.pdf]

Supplementary Figure S1

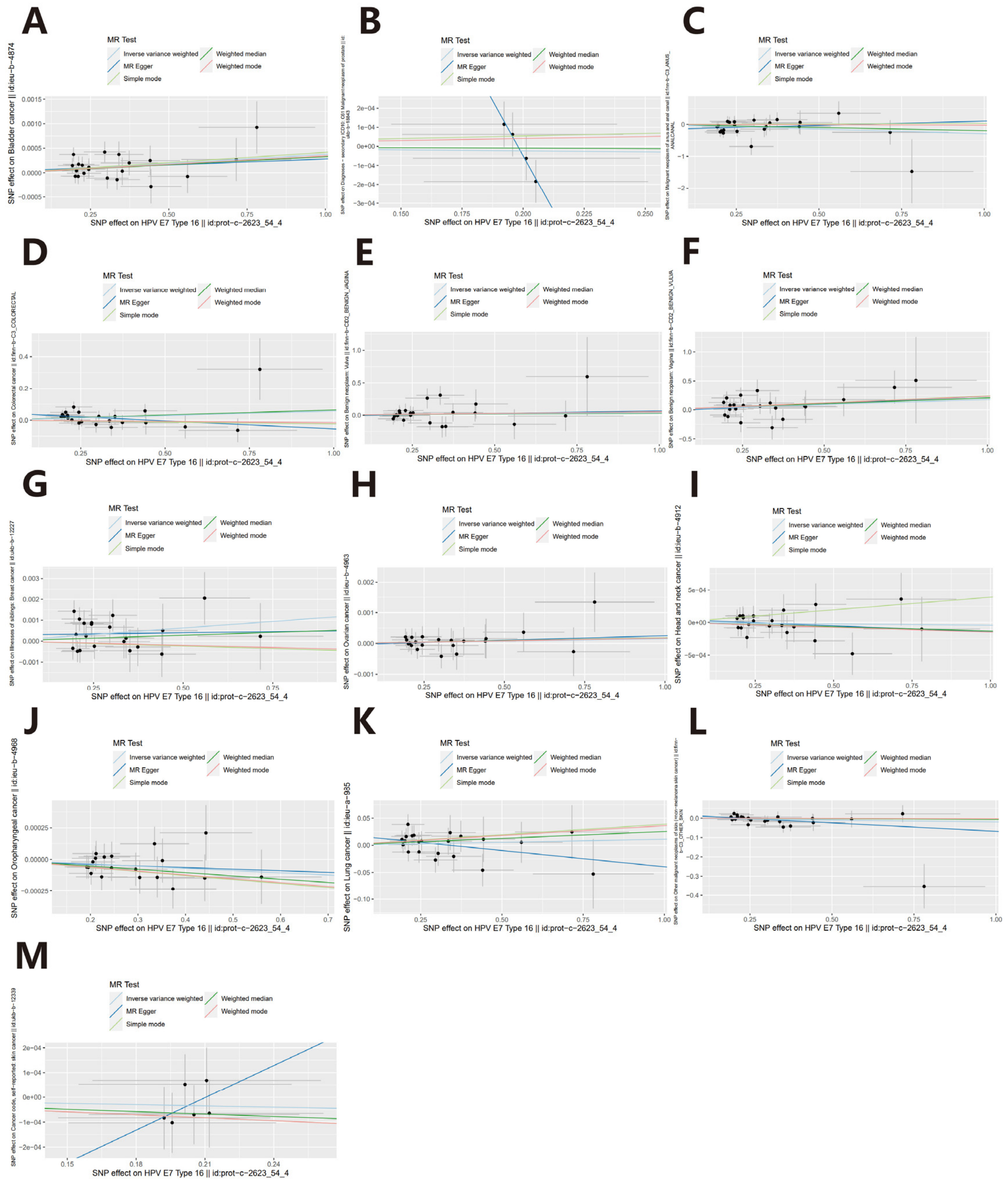

Figure S1. Scatter plots of SNPs associated with HPV 16 E7 protein and risk on (A) bladder cancer, (B) prostate cancer, (C) anal cancer, (D) colorectal cancer, (E) vaginal cancer, (F) vulvar cancer, (G) breast cancer, (H) ovarian cancer, (I) head and neck cancer, (J) oropharyngeal cancer, (K) lung cancer, (L) skin cancer (non−melanoma), (M) skin cancer incidence.

Supplementary Figure S2

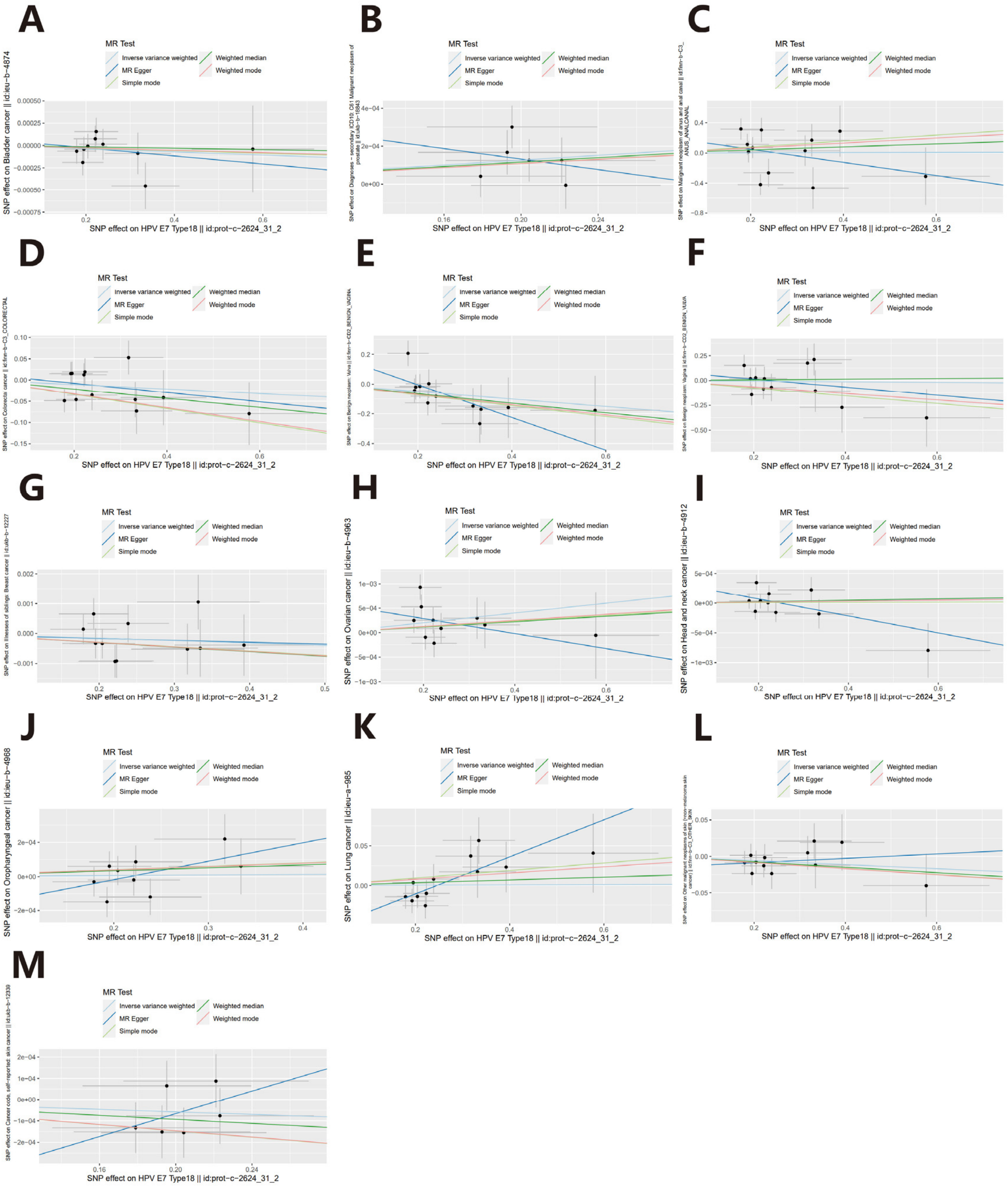

Figure S2. Scatter plots of SNPs associated with HPV 18 E7 protein and risk on (A) bladder cancer, (B) prostate cancer, (C) anal cancer, (D) colorectal cancer, (E) vaginal cancer, (F) vulvar cancer, (G) breast cancer, (H) ovarian cancer, (I) head and neck cancer, (J) oropharyngeal cancer, (K) lung cancer, (L) skin cancer (non−melanoma), (M) skin cancer incidence.

Supplementary Figure S3

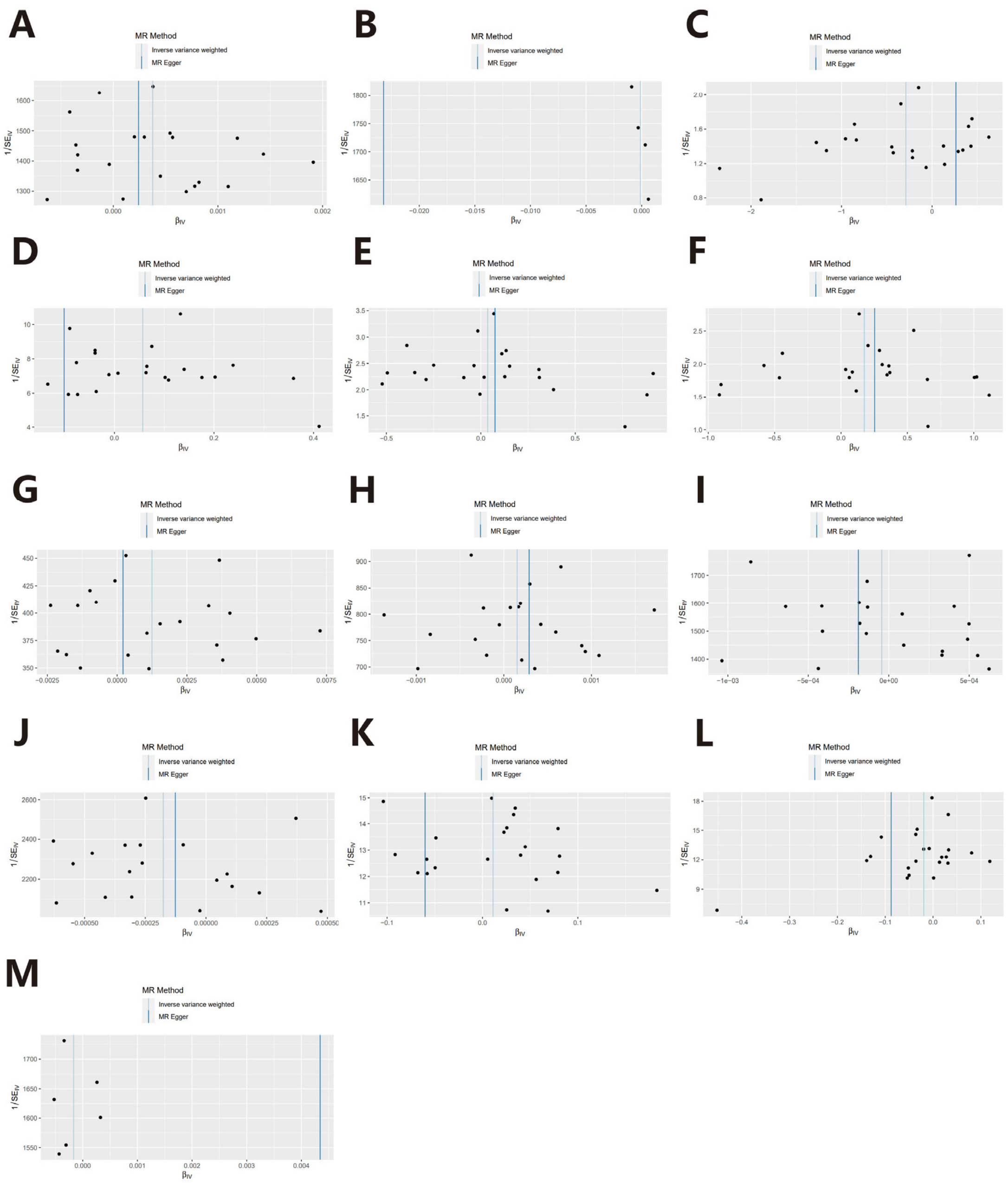

Figure S3. Funnel plots of the relationship between the causal effect of HPV 16 E7 protein and site-specific cancers. (A) bladder cancer, (B) prostate cancer, (C) anal cancer, (D) colorectal cancer, (E) vaginal cancer, (F) vulvar cancer, (G) breast cancer, (H) ovarian cancer, (I) head and neck cancer, (J) oropharyngeal cancer, (K) lung cancer, (L) skin cancer (non−melanoma), (M) skin cancer incidence.

Supplementary Figure S4

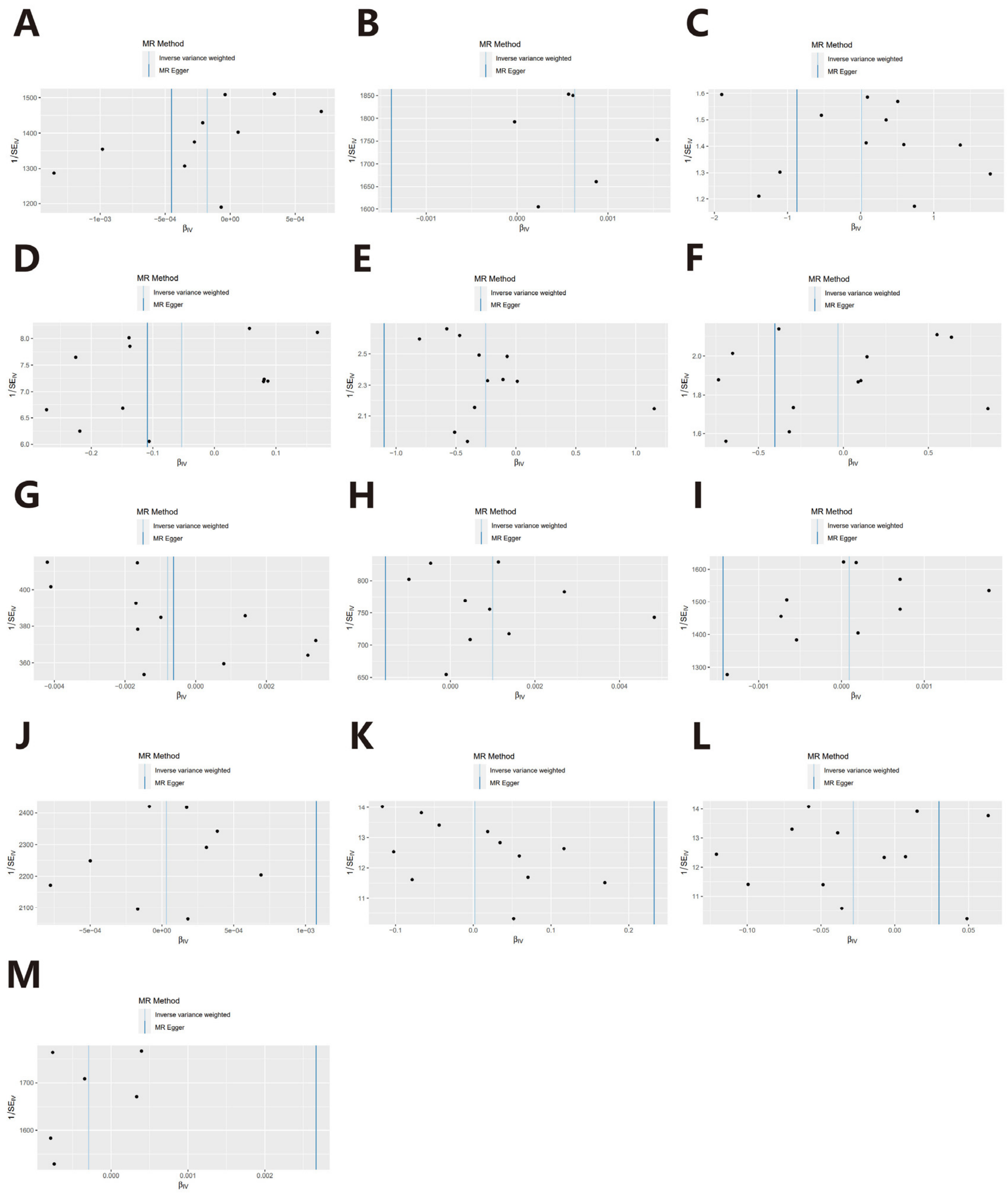

Figure S4. Funnel plots of the relationship between the causal effect of HPV 18 E7 protein and site-specific cancers. (A) bladder cancer, (B) prostate cancer, (C) anal cancer, (D) colorectal cancer, (E) vaginal cancer, (F) vulvar cancer, (G) breast cancer, (H) ovarian cancer, (I) head and neck cancer, (J) oropharyngeal cancer, (K) lung cancer, (L) skin cancer (non−melanoma), (M) skin cancer incidence.

Supplementary Figure S5

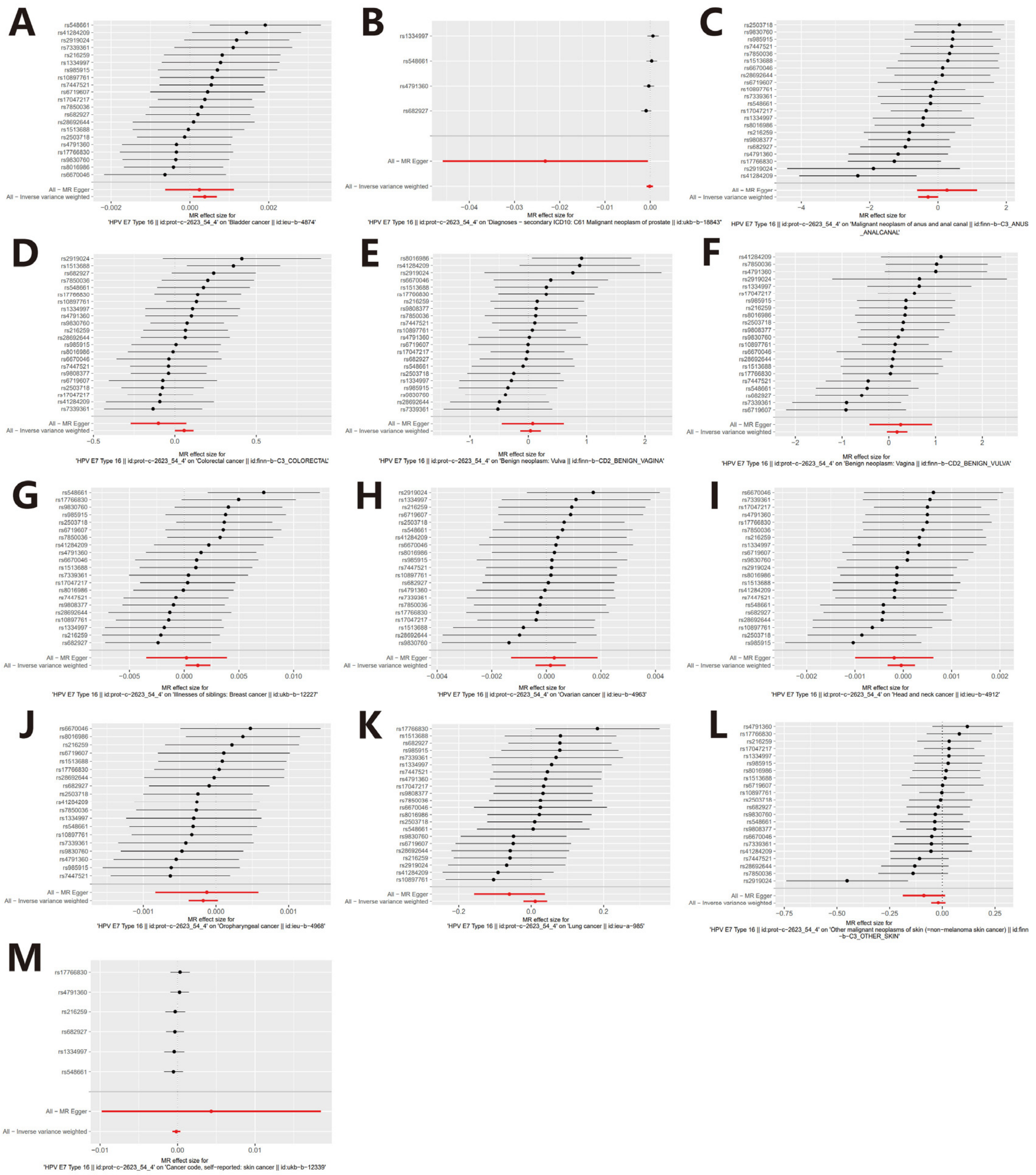

Figure S5. Forest plots of each SNP and total effect of HPV 16 E7 protein on (A) bladder cancer, (B) prostate cancer, (C) anal cancer, (D) colorectal cancer, (E) vaginal cancer, (F) vulvar cancer, (G) breast cancer, (H) ovarian cancer, (I) head and neck cancer, (J) oropharyngeal cancer, (K) lung cancer, (L) skin cancer (non−melanoma), (M) skin cancer incidence.

Supplementary Figure S6

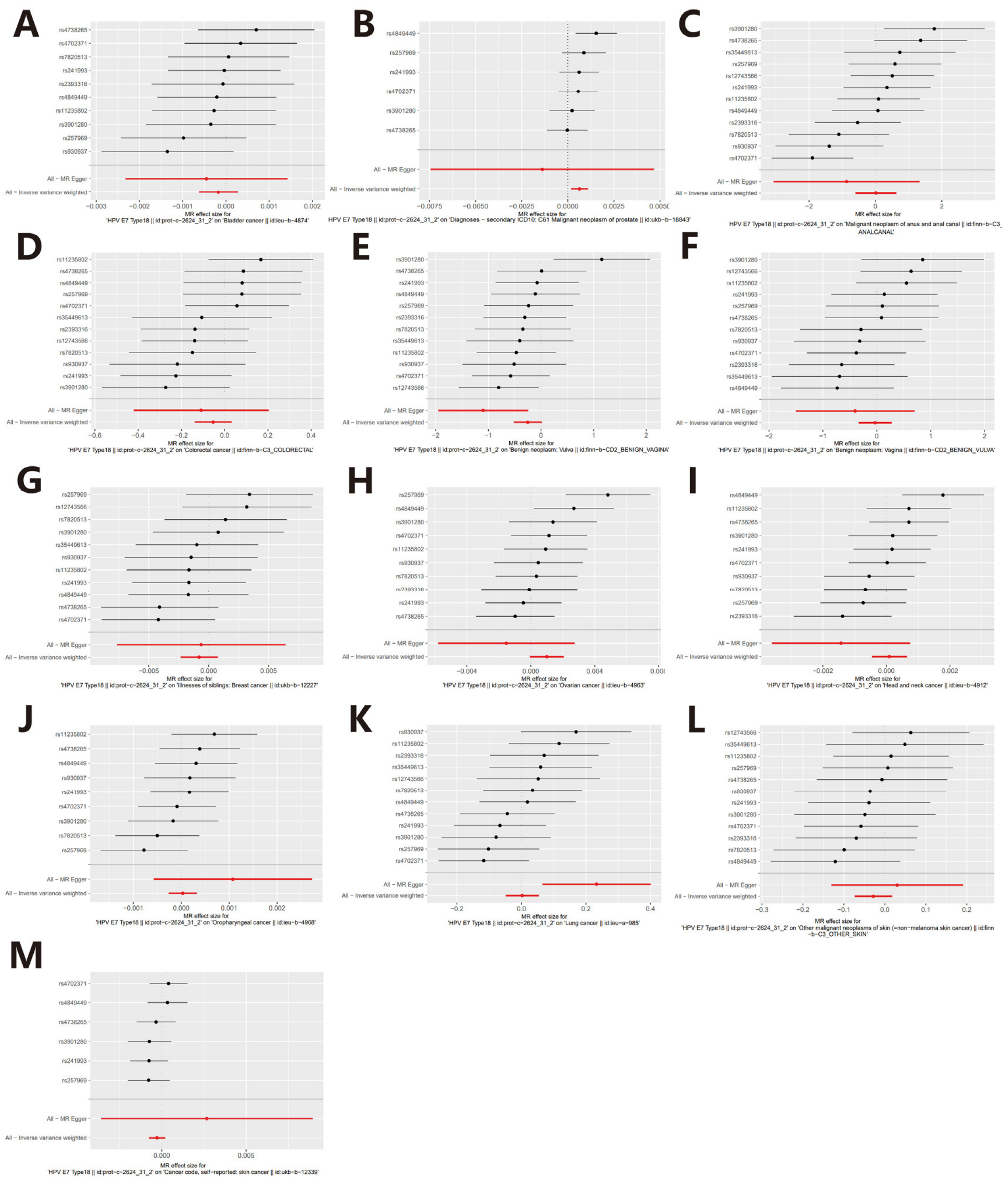

Figure S6. Forest plots of each SNP and total effect of HPV 18 E7 protein on (A) bladder cancer, (B) prostate cancer, (C) anal cancer, (D) colorectal cancer, (E) vaginal cancer, (F) vulvar cancer, (G) breast cancer, (H) ovarian cancer, (I) head and neck cancer, (J) oropharyngeal cancer, (K) lung cancer, (L) skin cancer (non−melanoma), (M) skin cancer incidence.

Supplementary Figure S7

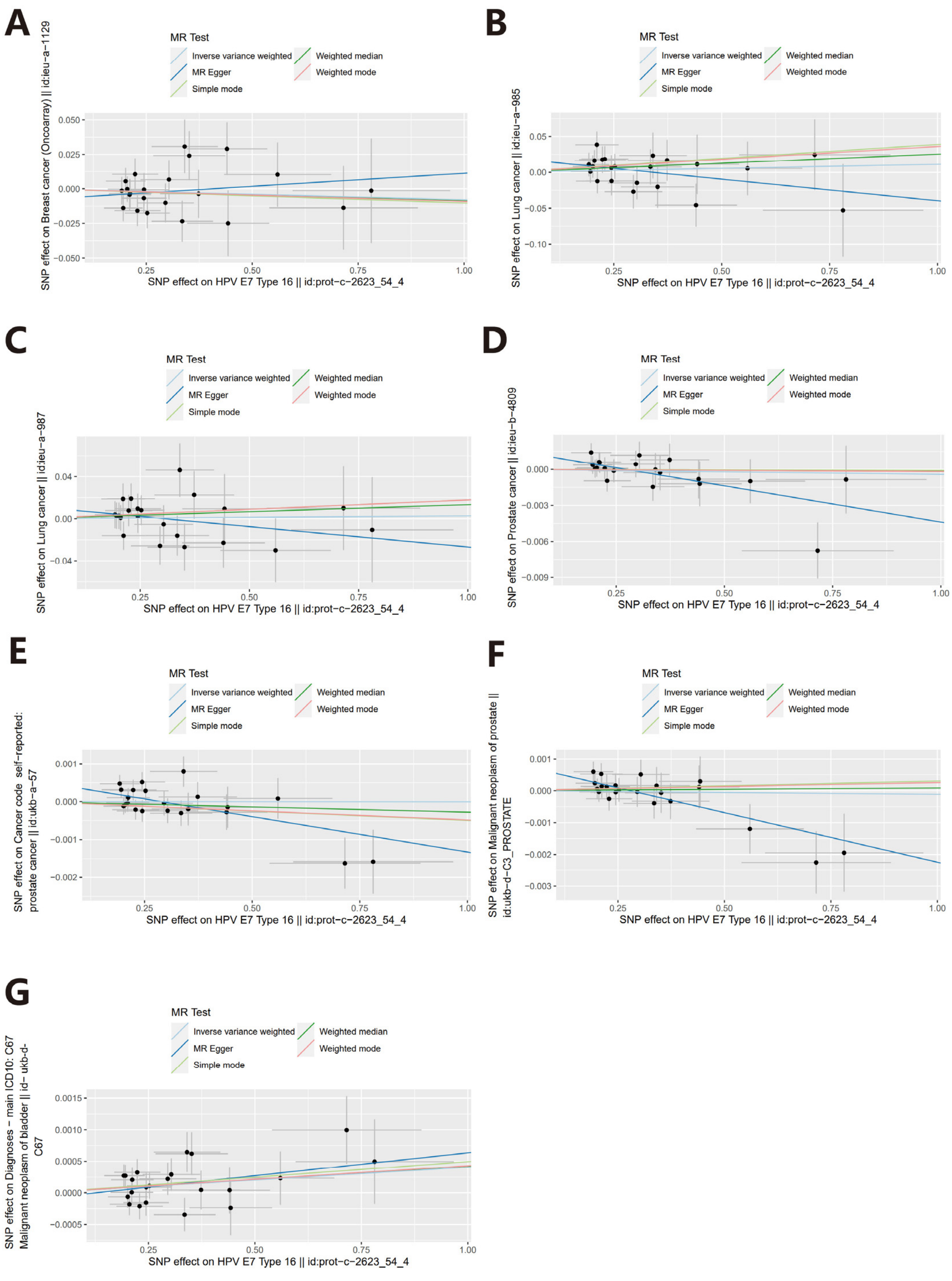

Figure S7. Scatter plots of SNPs associated with HPV 16 E7 protein and risk on (A) breast cancer; (B) lung cancer; (C-E) prostate cancer; (F) bladder cancer for validation.

Supplementary Figure S8

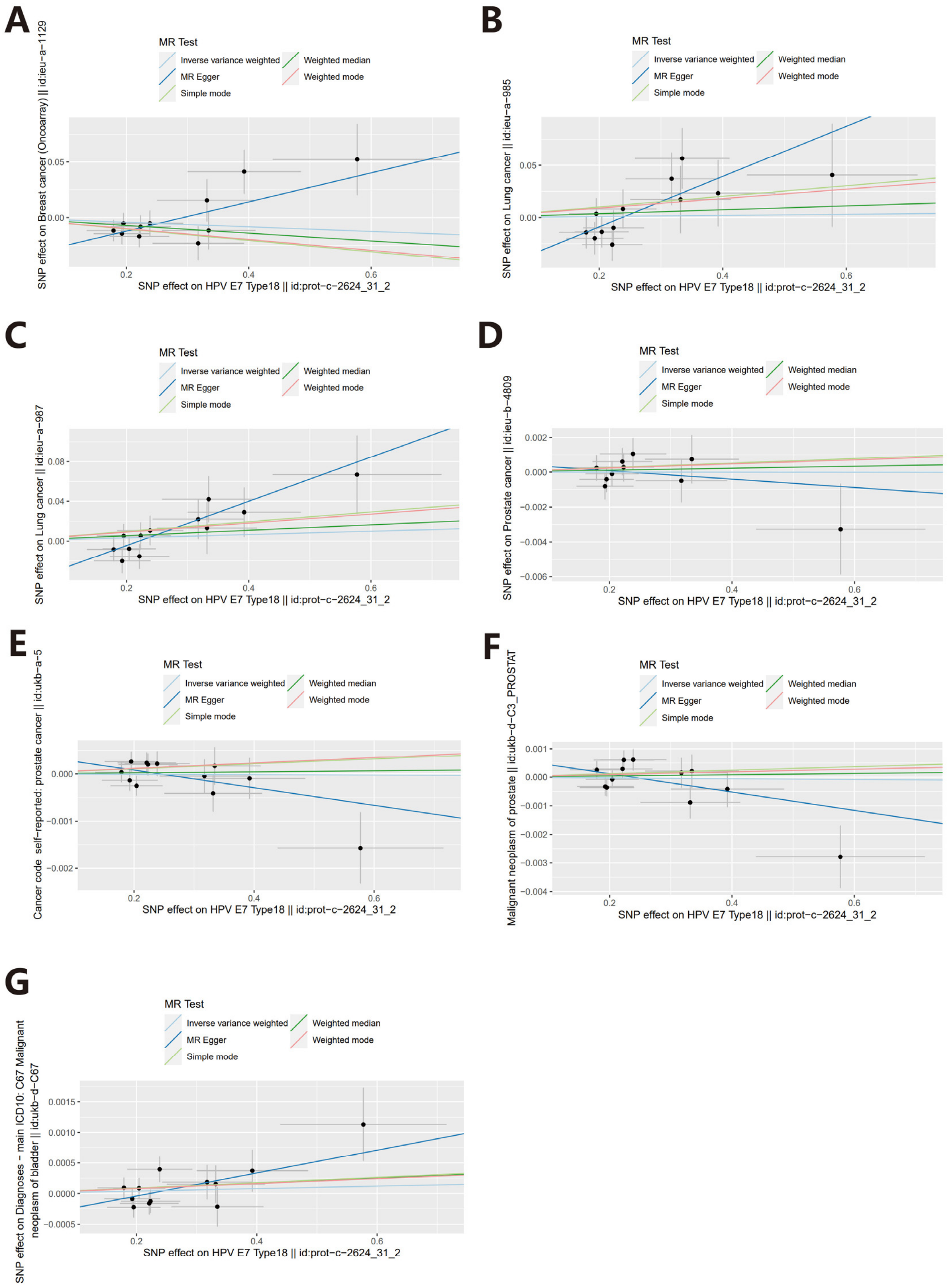

Figure S8. Scatter plots of SNPs associated with HPV 18 E7 protein and risk on (A) breast cancer; (B) lung cancer; (C-E) prostate cancer; (F) bladder cancer for validation.

Supplementary Figure S9

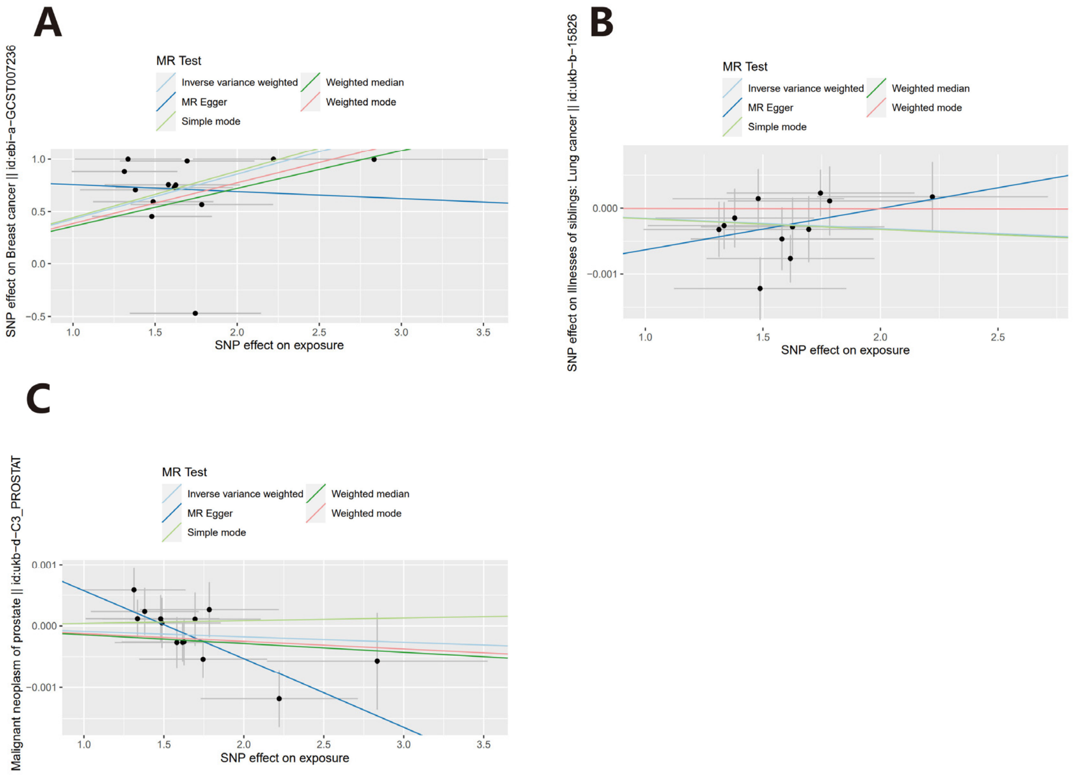

Figure S9. Scatter plots of SNPs associated with HPV chronic infection and risk on (A) breast cancer; (B) lung cancer; (C) prostate cancer for validation.

Supplementary Figure S10

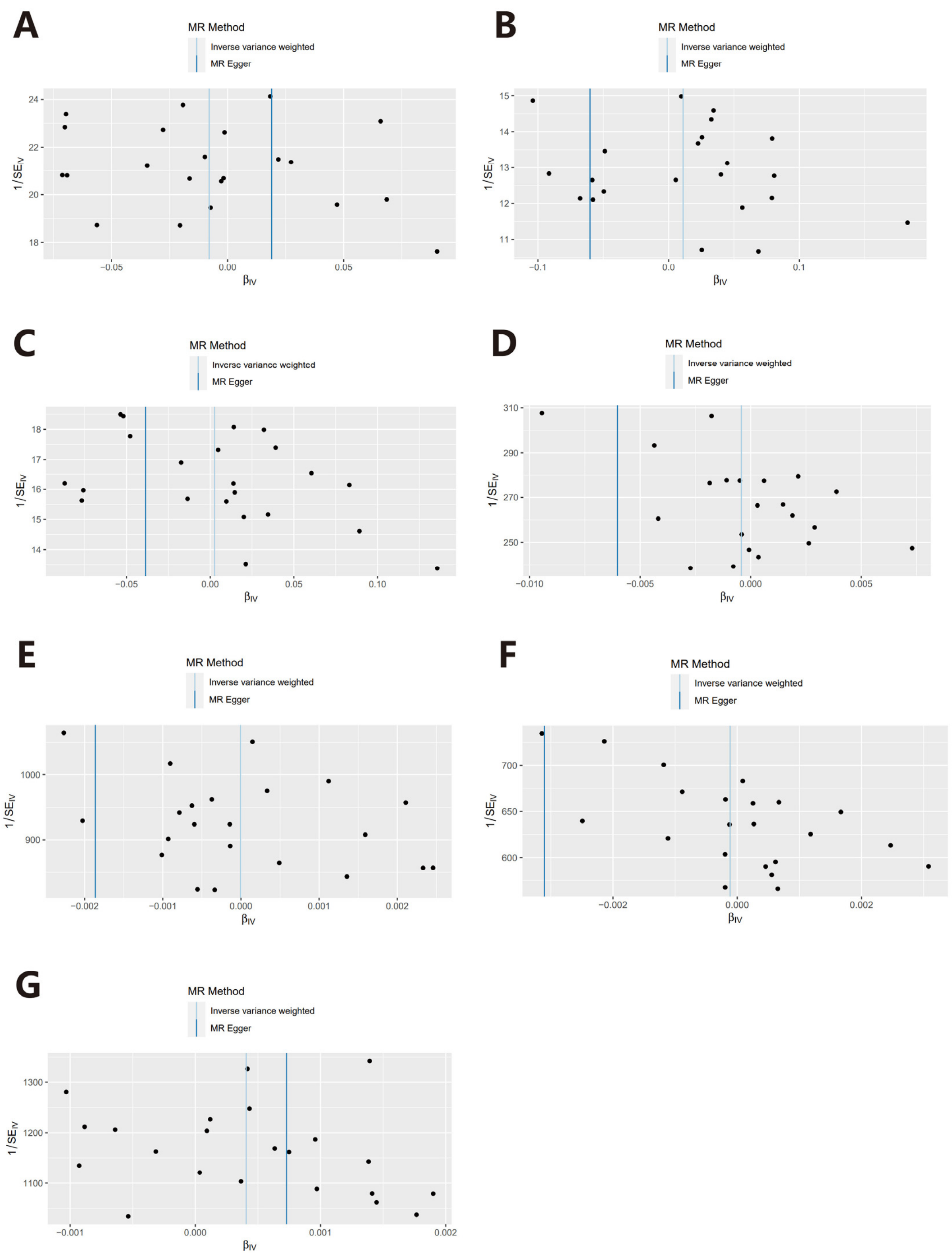

Figure S10. Funnel plots of the relationship between the causal effect of HPV 16 E7 protein and site-specific cancers for validation. (A) breast cancer; (B) lung cancer; (C-E) prostate cancer; (F) bladder cancer.

Supplementary Figure S11

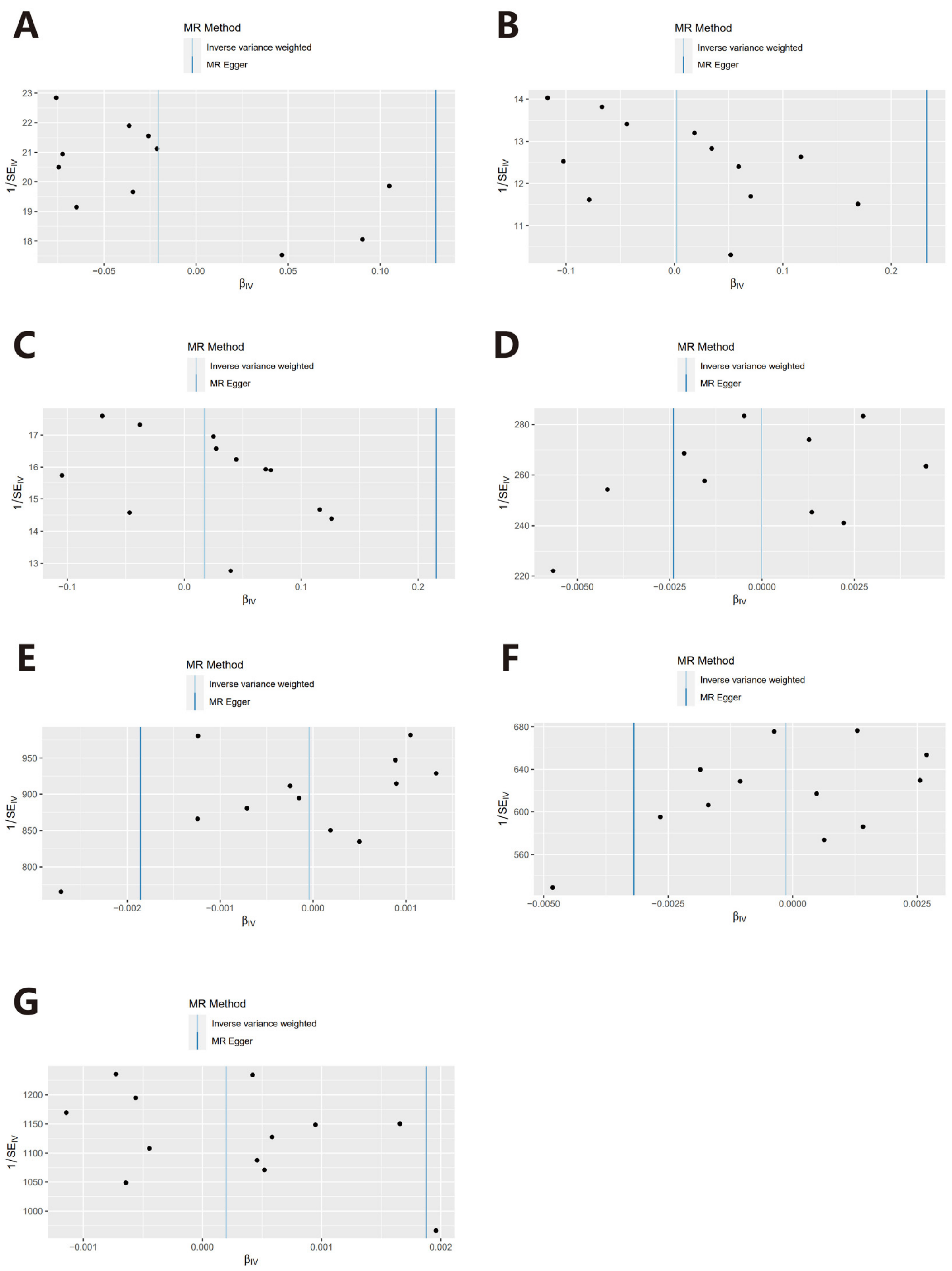

Figure S11. Funnel plots of the relationship between the causal effect of HPV 18 E7 protein and site-specific cancers for validation. (A) breast cancer; (B) lung cancer; (C-E) prostate cancer; (F) bladder cancer.

Supplementary Figure S12

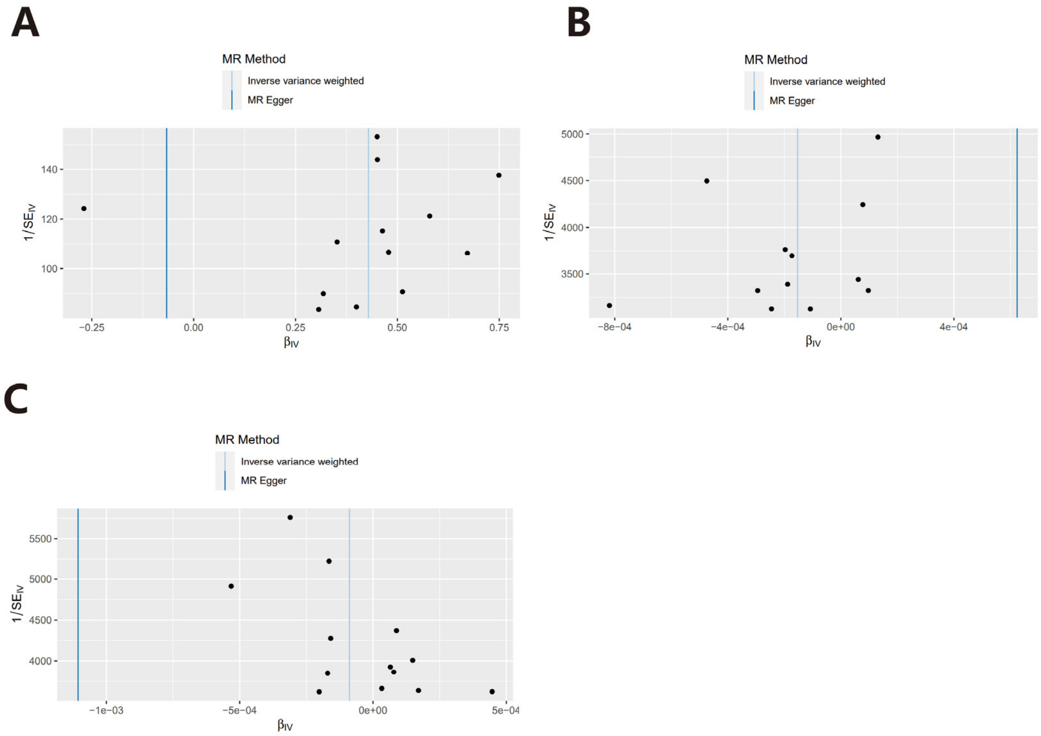

Figure S12. Funnel plots of the relationship between the causal effect of HPV chronic infection and site-specific cancers for validation. (A) breast cancer; (B) lung cancer; (C) prostate cancer for validation.

Supplementary Figure S13

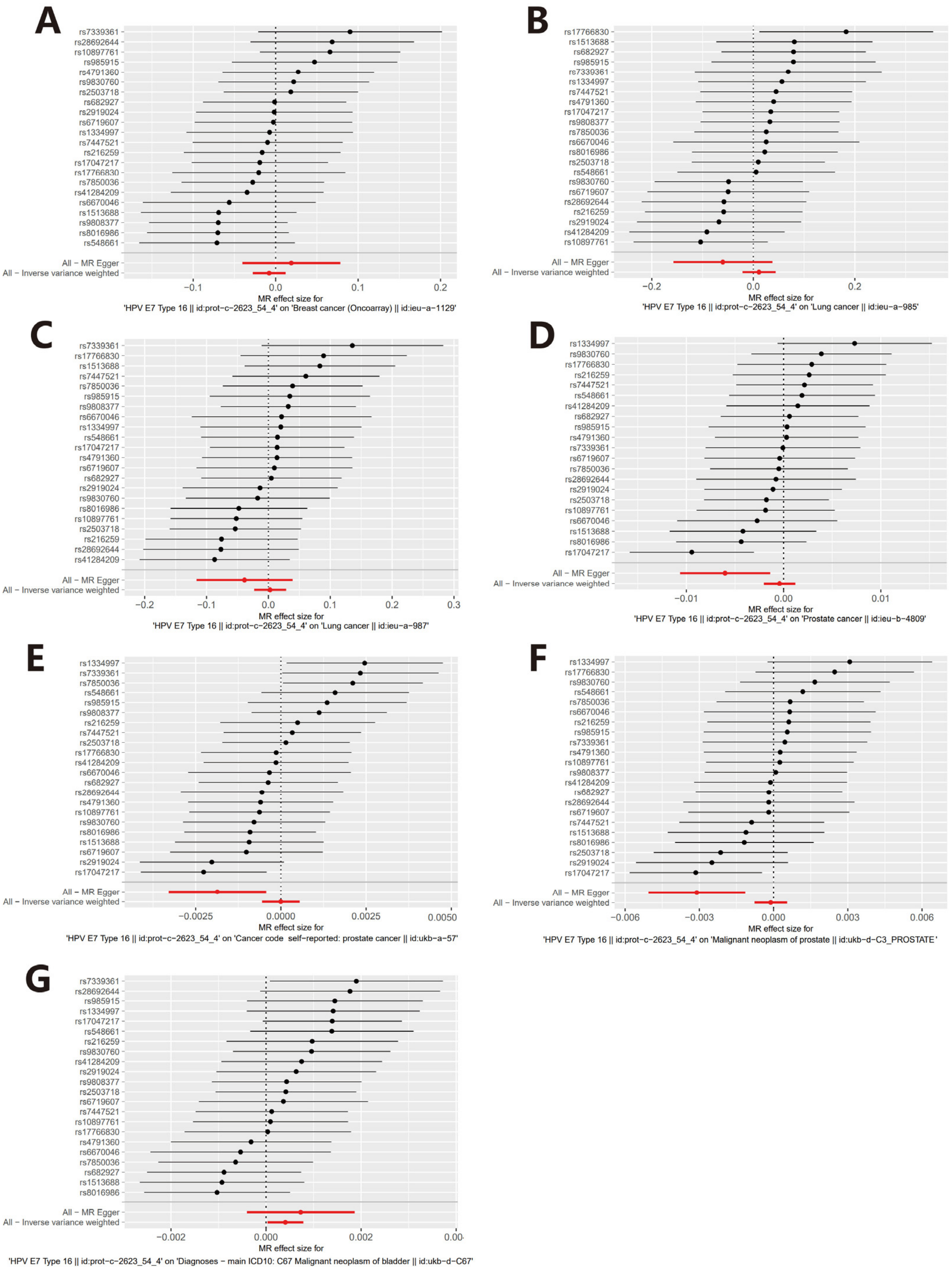

Figure S13. Forest plots of each SNP and total effect of HPV 16 E7 protein on (A) breast cancer; (B) lung cancer; (C-E) prostate cancer; (F) bladder cancer for validation.

Supplementary Figure S14

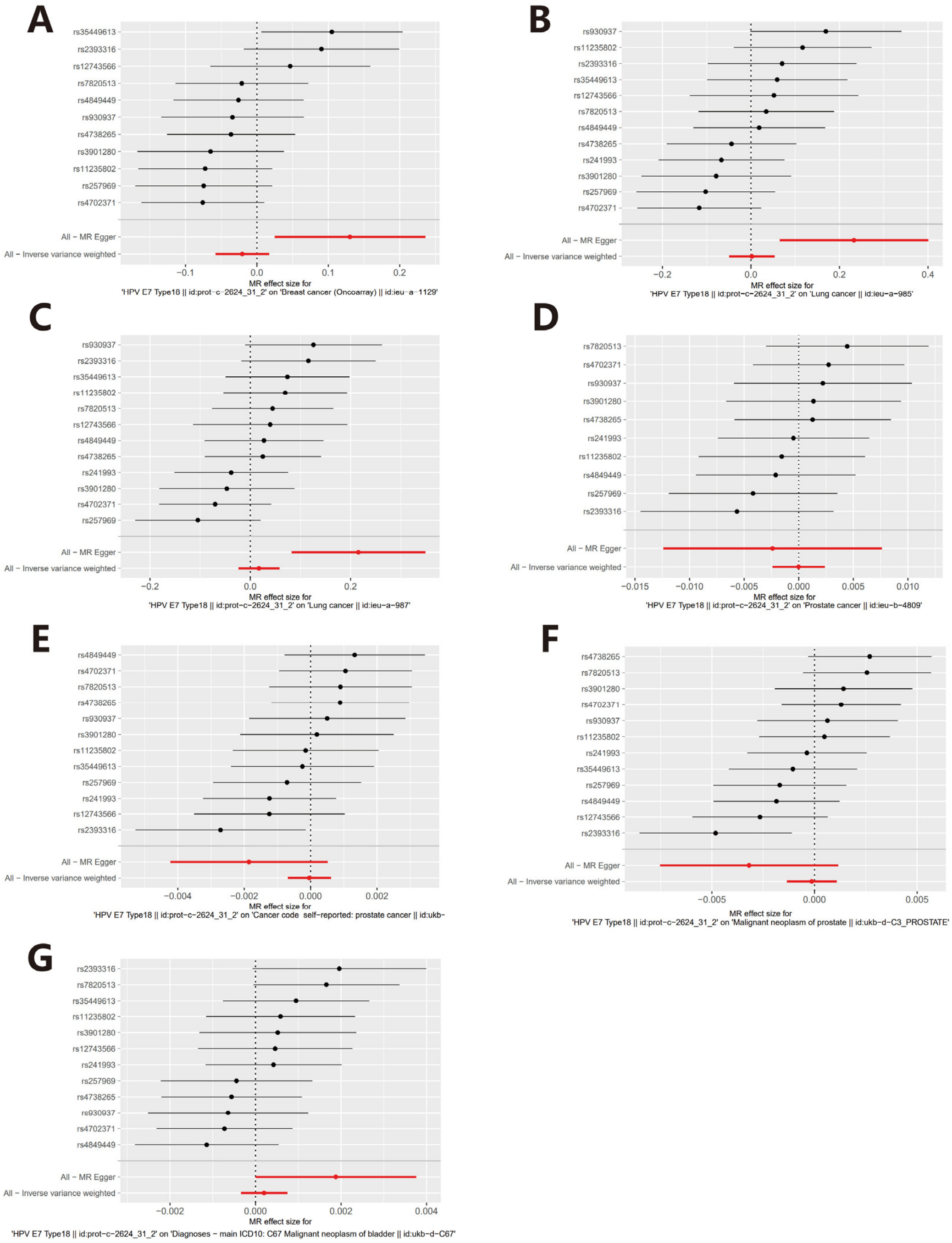

Figure S14. Forest plots of each SNP and total effect of HPV 18 E7 protein on (A) breast cancer; (B) lung cancer; (C-E) prostate cancer; (F) bladder cancer for validation.

Supplementary Figure S15

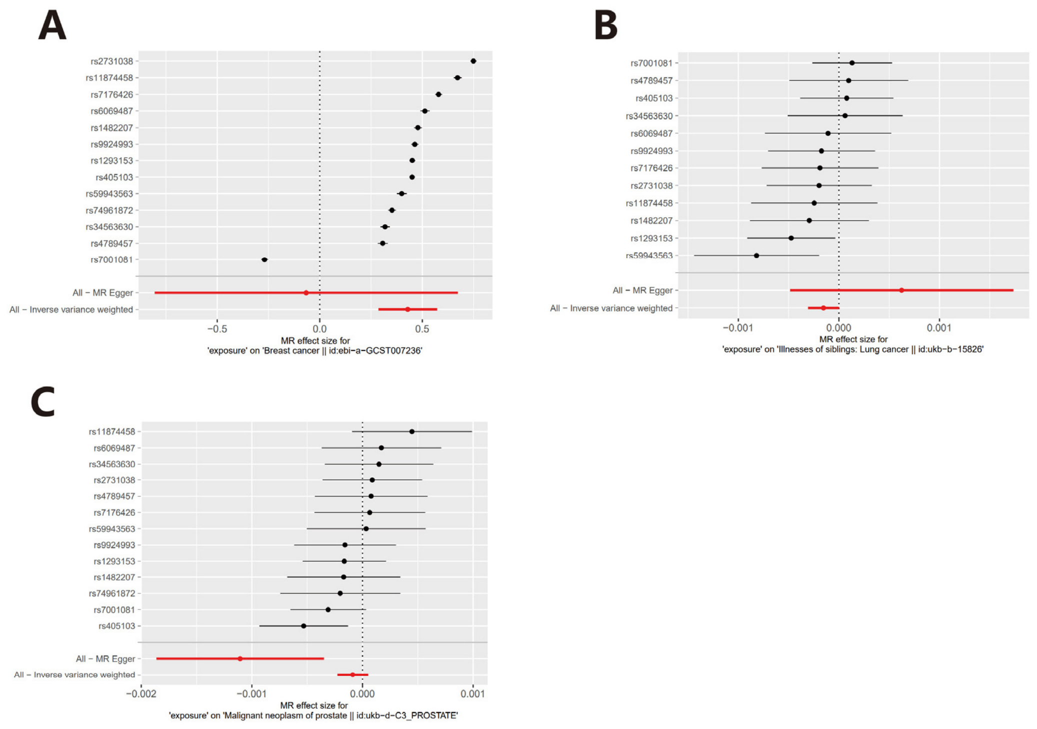

Figure S15. Forest plots of each SNP and total effect of HPV chronic infection on (A) breast cancer; (B) lung cancer; (C) prostate cancer for validation.

Supplementary Figure S16

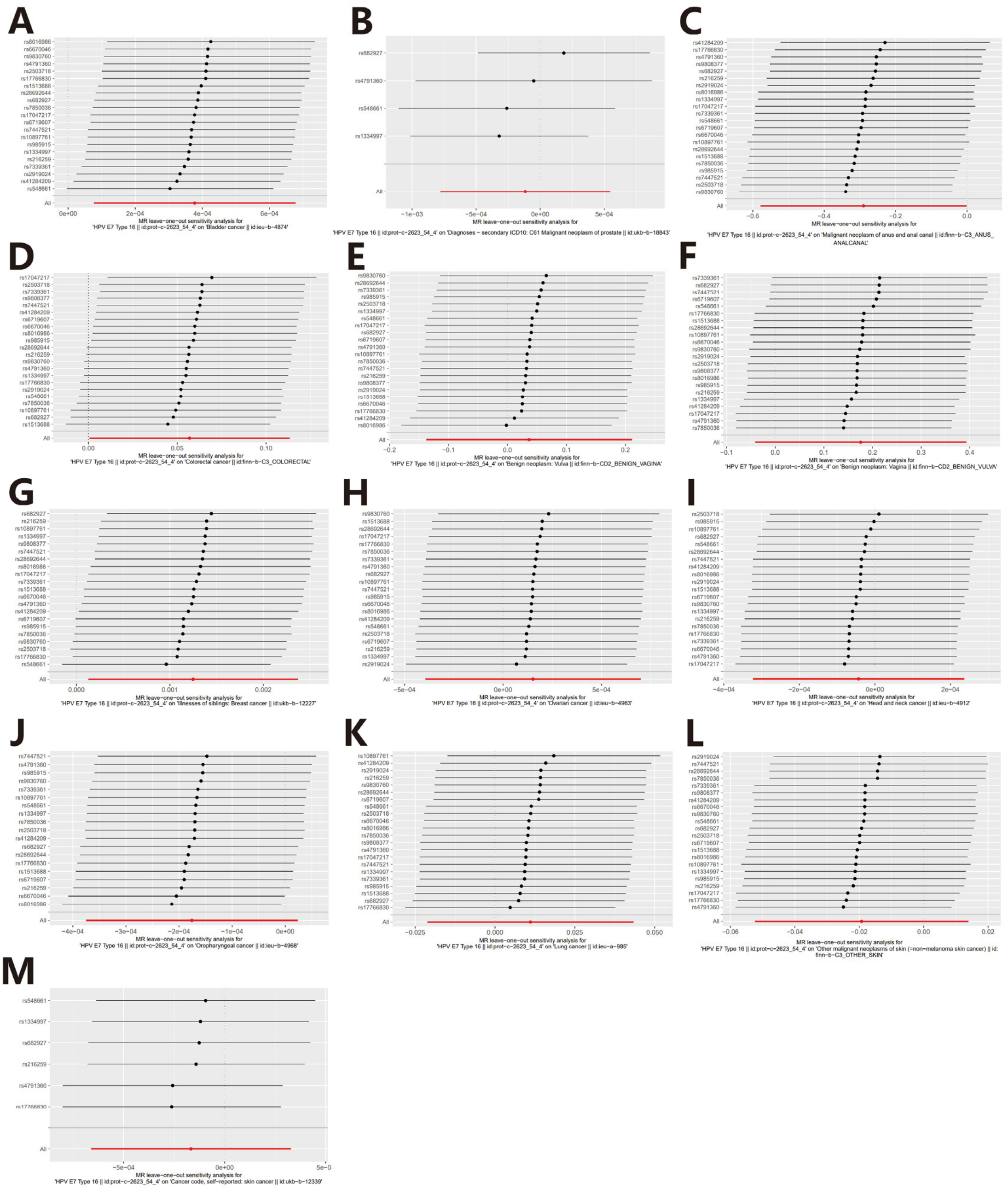

Figure S16. Leave-one-out analysis results of SNPs associated with HPV 16 E7 protein and risk on (A) bladder cancer, (B) prostate cancer, (C) anal cancer, (D) colorectal cancer, (E) vaginal cancer, (F) vulvar cancer, (G) breast cancer, (H) ovarian cancer, (I) head and neck cancer, (J) oropharyngeal cancer, (K) lung cancer, (L) skin cancer (non−melanoma), (M) skin cancer incidence.

Supplementary Figure S17

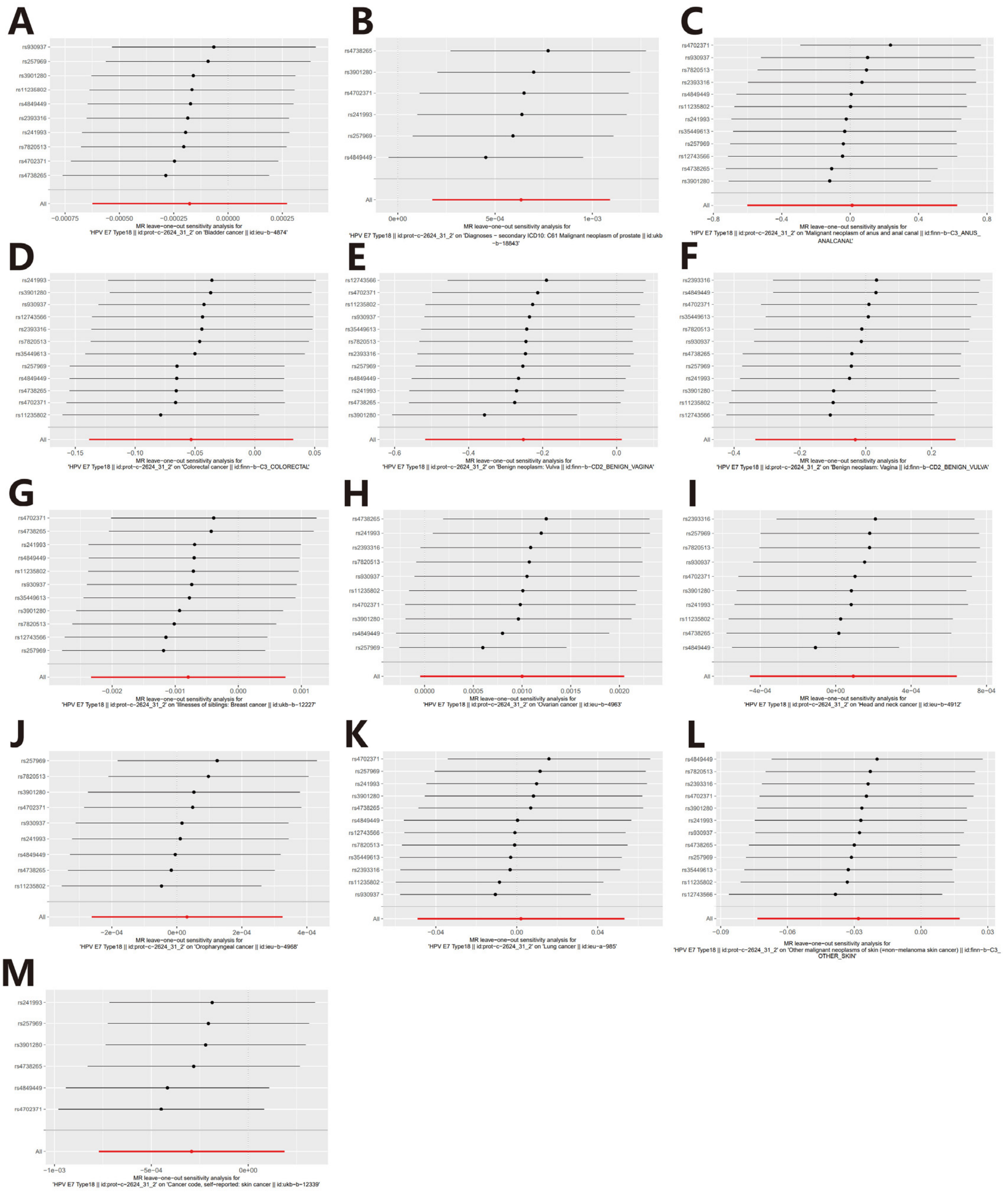

Figure S17. Leave-one-out analysis results of SNPs associated with HPV 18 E7 protein and risk on (A) bladder cancer, (B) prostate cancer, (C) anal cancer, (D) colorectal cancer, (E) vaginal cancer, (F) vulvar cancer, (G) breast cancer, (H) ovarian cancer, (I) head and neck cancer, (J) oropharyngeal cancer, (K) lung cancer, (L) skin cancer (non−melanoma), (M) skin cancer incidence.

Supplementary Figure S18

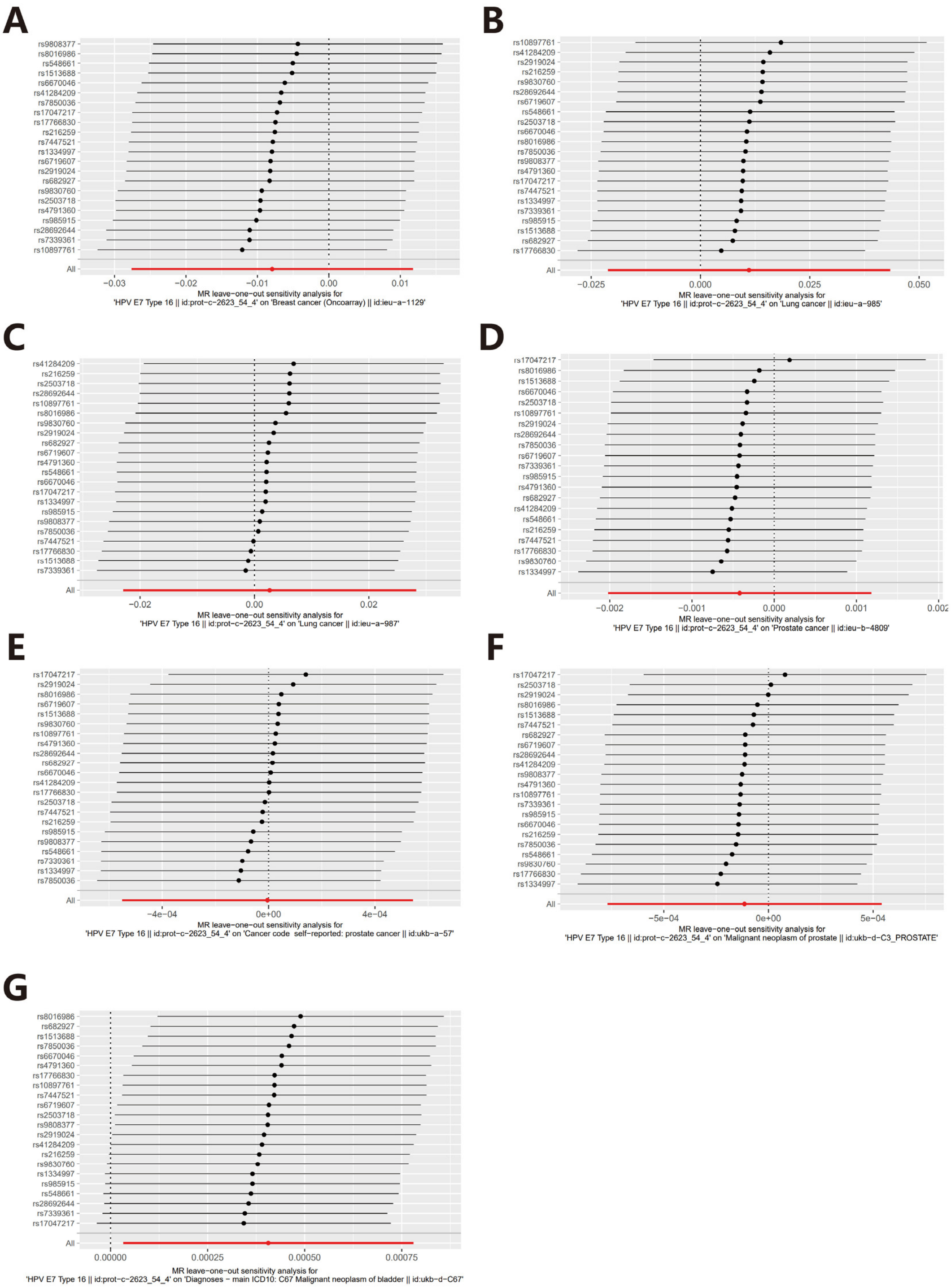

Figure S18. Leave-one-out analysis results of SNPs associated with HPV 16 E7 protein and risk on (A) breast cancer; (B) lung cancer; (C-E) prostate cancer; (F) bladder cancer incidence for validation.

Supplementary Figure S19

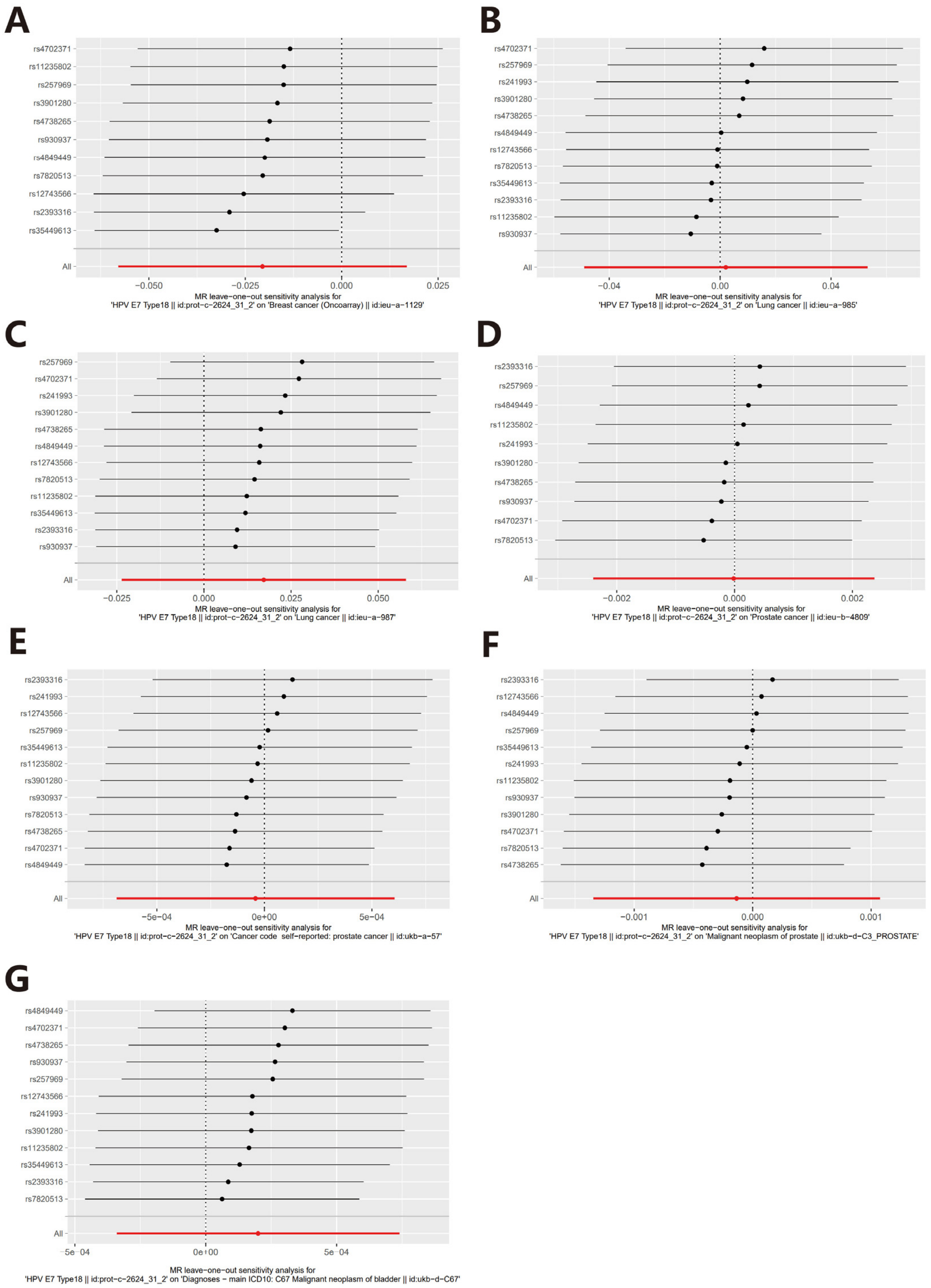

Figure S19. Leave-one-out analysis results of SNPs associated with HPV 18 E7 protein and risk on (A) breast cancer; (B) lung cancer; (C-E) prostate cancer; (F) bladder cancer incidence for validation.

Supplementary Figure S20

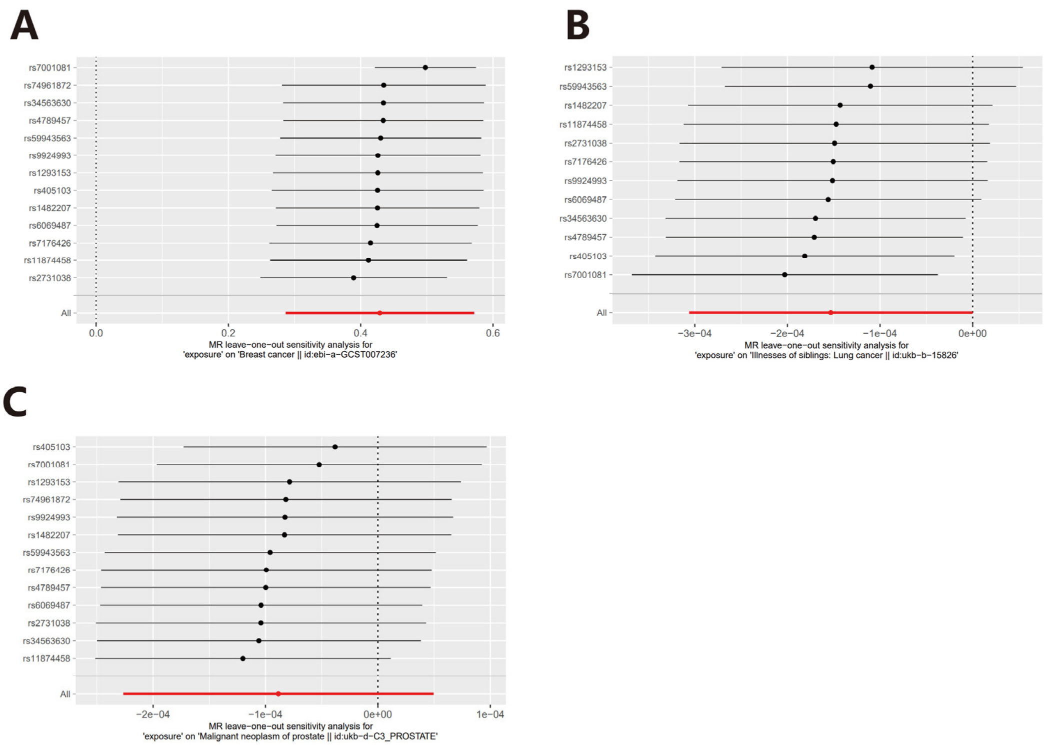

Figure S20. Leave-one-out analysis results of SNPs associated with HPV chronic infection and risk on (A) breast cancer; (B) lung cancer; (C) prostate cancer incidence for validation.
