# Supplementary material for: Unveiling the Association between HPV and Pan-Cancers: A Bidirectional Two-Sample Mendelian Randomization Study
Source: Cancers (Basel). 2023 Oct 26;15(21):5147. doi: 10.3390/cancers15215147 (PMC10650873; doi:10.3390/cancers15215147)
Supplement: Supplementary file 1 [file cancers-15-05147-s001.zip › Supplementary Tables.pdf]

Supplementary Table S1 | The detailed information of all statistical summary datasets used in this study

Usage purpose	Exposure/Outcome	Trait	GWAS ID / EFO ID	Year	Author	Population	Sample Size	n case	n control	n SNP
Univariate two-sample MR analysis & MR in Validation datasets	HPV 16 E7 Protein	HPV E7 Type 16	prot-c-2623_54_4	2017	Suhre K	European	997	NA	NA	501,428
	HPV 18 E7 Protein	HPV E7 Type18	prot-c-2624_31_2	2017	Suhre K	European	997	NA	NA	501,428
	Chronic HPV infection	chronic human papillomavirus infection	EFO_0010060	2019	Mainali B	Mix (Most white man)	191	40	151	737,742
	Bladder Cancer	Bladder cancer	ieu-b-4874	2021	Burrows	European	373,295	1,279	372,016	9,904,926
	Prostate Cancer	Diagnoses - secondary ICD10: C61 Malignant neoplasm of prostate	ukb-b-18843	2018	Ben Elsworth	European	463,010	1,309	461,701	9,851,867
	Anal Cancer	Malignant neoplasm of anus and anal canal	finn-b-C3_ANUS_ANALCANAL	2021	NA	European	218,792	108	218,684	16,380,466
	Colorectal Cancer	Colorectal cancer	finn-b-C3_COLORECTAL	2021	NA	European	218,792	3,022	215,770	16,380,466
	Vaginal Cancer	Benign neoplasm: Vagina	finn-b-CD2_BENIGN_VAGINA	2021	NA	European	123,579	297	123,282	16,379,784
Univariate two-sample MR analysis	Vulvar Cancer	Benign neoplasm: Vulva	finn-b-CD2_BENIGN_VULVA	2021	NA	European	123,579	192	123,387	16,379,784
	Breast Cancer	Illnesses of siblings: Breast cancer	ukb-b-12227	2018	Ben Elsworth	European	361,809	16,586	345,223	9,851,867
	Ovarian Cancer	Ovarian cancer	ieu-b-4963	2021	Burrows	European	199,741	1,218	198,523	9,822,229
	Head and Neck Cancer	Head and neck cancer	ieu-b-4912	2021	Burrows	European	373,122	1,106	372,016	9,655,080
	Oropharyngeal Cancer	Oropharyngeal cancer	ieu-b-4968	2021	Burrows	European	372,510	494	372,016	8,283,869
	Lung Cancer	Lung cancer	ieu-a-985	NA	NA	European	40,453	23,848	16,605	7,877,791
	Skin Cancer	Other malignant neoplasms of skin (=non-melanoma skin cancer)	finn-b-C3_OTHER_SKIN	2021	NA	European	218,410	10,382	208,410	16,380,466
	Skin Cancer	Cancer code, self-reported: skin cancer	ukb-b-12339	2018	Ben Elsworth	European	462,933	1,436	461,497	9,851,867
	Bladder Cancer	Diagnoses - main ICD10: C67 Malignant neoplasm of bladder	ukb-d-C67	2018	Neale lab	European	361,194	1,554	359,640	10,267,743
	Prostate Cancer	Malignant neoplasm of prostate	ukb-d-C3_PROSTATE	2018	Neale lab	European	361,194	6,321	354,873	12,453,833
	Prostate Cancer	Cancer code self-reported: prostate cancer	ukb-a-57	2017	Neale Lab	European	337,159	2,495	334,664	10,894,596
Validation MR analysis	Prostate Cancer	Prostate cancer	ieu-b-4809	2021	Burrows	European	182,625	9,132	173,493	NA
	Breast Cancer	Breast cancer	ebi-a-GCST007236	2015	Michailidou K	European	89,677	46,785	42,892	13,742,658
	Breast Cancer	Breast cancer (Oncoarray)	ieu-a-1129	2017	Michailidou K	European	106,776	61,282	45,494	10,680,257
	Lung Cancer	Lung cancer	ieu-a-987	NA	NA	European	85,449	29,863	55,586	10,439,018
	Lung Cancer	Illnesses of siblings: Lung cancer	ukb-b-15826	2018	Ben Elsworth	European	361,586	8,199	353,387	9,851,867

Supplementary Table S2 | The information of all SNPs from HPV16 E7 protein dataset

SNP	Trait & Source	Gene	Position (hg19)	Function	R^2	F-Statistics
rs1334997	NA	LINC01788	chr1:71154117	intergenic	0.017	17.31
rs548661	NA	GRIK3	chr1:37479392	intron	0.019	18.74
rs6670046	NA	RP11-563D10.1	chr1:194104179	intergenic	0.021	20.97
rs2919024	Cause of death: multisystem degeneration (UKBB); IGP16 (PMID: 23382691); IGP20 (PMID: 23382691); IgG glycosylation (PMID: 23382691)	PLD5	chr1:242522042	intron	0.018	17.70
rs9808377	Pulse rate (UKBB)	TTN	chr2:179421694	missense	0.022	22.86
rs6719607	Arm fat percentage left (UKBB); Body mass index (UKBB)	RN7SL361P	chr2:60866057	upstream	0.017	17.22
rs17047217	Self-reported liver or hepatocellular cancer (UKBB)	PNPT1	chr2:55970893	intergenic	0.017	16.67
rs9830760	NA	CACNA2D3	chr3:54264197	intron	0.020	20.46
rs2864426	NA	SIAH2	chr3:145529197	intergenic	0.017	17.15
rs7447521	NA	CTD-2197M16.1	chr5:27610789	intergenic	0.017	16.91
rs2503718	Height (UKBB)	RP11-207F8.1	chr6:81380831	intergenic	0.019	19.67
rs985915	NA	EYS	chr6:65433784	intron	0.017	17.20
rs216259	NA	FAM65B	chr6:24931083	intron	0.018	18.20
rs682927	NA	RNA5SP279	chr9:1358244	intergenic	0.020	20.03
rs7850036	NA	ANKRD19P	chr9:95589160	intron	0.021	21.24
rs10897761	Cause of death: home (UKBB)	RP11-170L9.1	chr11:80884316	intergenic	0.021	21.70
rs7339361	NA	MYO16	chr13:109504132	intron	0.019	19.10
rs28692644	NA	CAB39L	chr13:50005050	intron	0.017	17.20
rs41284209	NA	MED4	chr13:48650492	3_prime_UTR	0.020	19.71
rs8016986	NA	LINC01148	chr14:87365603	intergenic	0.021	21.50
rs4791360	NA	GSG1L2	chr17:9713505	intron	0.019	18.82
rs1513688	NA	RP11-244M2.1	chr18:37304602	intron	0.018	17.92
rs17766830	IgG galactosylation (PMID: 28878392); Overall health rating (UKBB)	RNF165	chr18:44040660	3_prime_UTR	0.018	17.94
Average F-Statistic	-	-	-	-	-	18.97

Supplementary Table S3 | The information of all SNPs from HPV18 E7 protein dataset

SNP	Trait & Source	Gene	Position (hg19)	Function	R^2	F-Statistics
rs12743566	Frequency of tiredness or lethargy in last 2 weeks (UKBB)	SLC44A5	chr1:75700632	intron	0.016	16.60
rs35449613	NA	DPT	chr1:168714238	Missense	0.018	18.03
rs4849449	NA	DPP10	chr2:116955767	intergenic	0.019	19.54
rs930937	NA	DPP3P1	chr4:58935390	intergenic	0.019	19.14
rs249011	Height (UKBB); Qualifications: college or university degree (UKBB); Trunk fat-free mass (UKBB); Trunk predicted mass (UKBB); Treatment with bendrofluazide (UKBB)	FAM151B	chr5:79809658	NA	0.020	19.67
rs257969	NA	HSD17B4	chr5:118837658	intron	0.017	17.35
rs4702371	NA	NSUN2	chr5:6621031	intron	0.020	20.72
rs4738265	Weight (UKBB); Waist circumference (UKBB); Leg fat mass left (UKBB); Body mass index (UKBB); Whole body fat mass (UKBB)	KCNB2	chr8:73443576	intergenic	0.020	20.62
rs7820513	NA	LINC01419	chr8:84495551	intergenic	0.019	19.51
rs2393316	Cause of death: creutzfeldt-jakob disease (UKBB); Treatment with ventolin 100micrograms inhaler (UKBB); Self-reported meningitis (UKBB)	RP11-550A9.1	chr10:59333071	intergenic	0.017	17.47
rs11235802	NA	FAM168A	chr11:73259543	intron	0.018	18.10
rs241993	NA	CRACR2A	chr12:3818665	intron	0.022	22.24
rs3901280	Leg fat percentage left (UKBB); Leg fat mass left (UKBB)	RP11-483K5.2	chr16:8510914	intergenic	0.017	16.71
Average F-Statistic	-	-	-	-	-	18.90

Supplementary Table S4 | The information of all SNPs from HPV chronic infection dataset

SNP	Trait & Source	Gene	Position (hg19)	Function	R^2	F-Statistics
rs34563630	Leg fat percentage left (UKBB); Leg fat mass left (UKBB)	DPP3P1	chr4:61849734	intergenic	0.082	16.80
rs1482207	NA	CSMD1	chr8:3503182	intron	0.080	16.43
rs74961872	Cause of death: other specified place (UKBB)	RP11-546K22.1	chr8:52968730	intergenic	0.081	16.74
rs7001081	Systolic blood pressure (UKBB)	LINC01289	chr8:64685833	intron	0.091	18.96
rs2731038	NA	DBX2	chr12:45417666	Missense	0.082	16.77
rs7176426	NA	SLC12A6	chr15:34614257	intron	0.083	17.04
rs405103	NA	RP11-326A19.4	chr15:89617230	intron	0.097	20.31
rs9924993	NA	U91319.1	chr16:13639660	intron	0.084	17.23
rs4789457	NA	RP11-13K12.2	chr17:75516811	intergenic	0.080	16.34
rs59943563	NA	PTPRM	chr18:8266569	intron	0.080	16.40
rs11874458	Body mass index (PMID: 28892062); Cholelithiasis (UKBB); Self-reported respiratory infection (UKBB); Acute pharyngitis (UKBB)	DCC	chr18:50572367	intron	0.081	16.57
rs1293153	NA	RP13-379L11.3	chr20:52951047	intergenic	0.097	20.37
rs6069487	NA	RP13-379L11.3	chr20:54515066	intergenic	0.081	16.63
Average F-Statistic	-	-	-	-	-	17.43

Supplementary Table S5 | The results of reverse MR analysis

Exposure and methods		Outcomes			
		HPV16 E7		HPV18 E7	
Main.exposure	Method	nSNP	P.value	nSNP	P.value
Bladder Cancer	MR Egger	10	0.414	10	0.718
(ieu-b-4874)	Weighted median	10	0.345	10	0.738
	Inverse variance weighted	10	0.320	10	0.347
	Simple mode	10	0.563	10	0.824
	Weighted mode	10	0.512	10	0.967
Anal Cancer	MR Egger	4	0.369	4	0.940
(finn-b-C3_ANUS_ANALCANAL)	Weighted median	4	0.353	4	0.667
	Inverse variance weighted	4	0.279	4	0.668
	Simple mode	4	0.642	4	0.832
	Weighted mode	4	0.436	4	0.624
Prostate Cancer	MR Egger	2	NA	2	NA
(ukb-b-18843)	Weighted median	2	NA	2	NA
	Inverse variance weighted	2	NA	2	0.121
	Simple mode	2	NA	2	NA
	Weighted mode	2	NA	2	NA
Vaginal Cancer	MR Egger	10	0.066	10	0.426
(finn-b-CD2_BENIGN_VAGINA)	Weighted median	10	0.225	10	0.384
	Inverse variance weighted	10	0.645	10	0.843
	Simple mode	10	0.514	10	0.354
	Weighted mode	10	0.204	10	0.327
Breast Cancer	MR Egger	19	0.153	19	0.999
(ukb-b-12227)	Weighted median	19	0.271	19	0.228
	Inverse variance weighted	19	0.386	19	0.446
	Simple mode	19	0.652	19	0.430
	Weighted mode	19	0.477	19	0.306
Colorectal Cancer	MR Egger	15	0.170	15	0.039
(finn-b-C3_COLORECTAL)	Weighted median	15	0.764	15	0.601
	Inverse variance weighted	15	0.937	15	0.877
	Simple mode	15	0.646	15	0.574
	Weighted mode	15	0.712	15	0.712
Lung Cancer	MR Egger	19	0.474	19	0.525
(ieu-a-985)	Weighted median	19	0.364	19	0.853
	Inverse variance weighted	19	0.141	19	0.830
	Simple mode	19	0.544	19	0.443
	Weighted mode	19	0.524	19	0.459
Vulvar Cancer	MR Egger	4	0.658	4	0.658
(finn-b-CD2_BENIGN_VULVA)	Weighted median	4	0.481	4	0.481
	Inverse variance weighted	4	0.541	4	0.541
	Simple mode	4	0.555	4	0.555
	Weighted mode	4	0.412	4	0.412
Oropharyngeal Cancer	MR Egger	5	0.864	5	0.335
(ieu-b-4968)	Weighted median	5	0.746	5	0.409
	Inverse variance weighted	5	0.231	5	0.479
	Simple mode	5	0.875	5	0.441

	Weighted mode	5	0.867	5	0.422
Head and Neck Cancer (ieu-b-4912)	MR Egger	12	0.306	12	0.151
	Weighted median	12	0.934	12	0.090
	Inverse variance weighted	12	0.752	12	0.239
	Simple mode	12	0.758	12	0.191
	Weighted mode	12	0.731	12	0.195
Ovarian Cancer (ieu-b-4963)	MR Egger	9	0.168	9	0.634
	Weighted median	9	0.347	9	0.109
	Inverse variance weighted	9	0.150	9	0.055
	Simple mode	9	0.535	9	0.182
	Weighted mode	9	0.556	9	0.157
Skin Cancer (non-melanoma) (finn-b-C3_OTHER_SKIN)	MR Egger	18	0.260	18	0.671
	Weighted median	18	0.475	18	0.443
	Inverse variance weighted	18	0.848	18	0.981
	Simple mode	18	0.870	18	0.629
	Weighted mode	18	0.337	18	0.336
Skin Cancer (ukb-b-12339)	MR Egger	1	NA	1	NA
	Weighted median	1	NA	1	NA
	Inverse variance weighted	1	NA	1	NA
	Simple mode	1	NA	1	NA
	Weighted mode	1	NA	1	NA
